# Oncolytic myxoma virus and engineering strategies for advanced cancer immunotherapy

**DOI:** 10.1016/j.isci.2026.115812

**Published:** 2026-04-20

**Authors:** A.D. Trujillo Yeriomenko, A.S. Isaeva, E.E. Idota, S.P. Zhivoderov, S.G. Yurkov, A.S. Malogolovkin

**Affiliations:** 1 First Moscow State Medical University (Sechenov University), Moscow 119048, Russia; 2 Moscow Institute of Physics and Technology, Dolgoprudny, Moscow oblast 141700, Russia; 3 Federal Research Center for Virology and Microbiology, Academician Bakoulov Street, Bldg. 1, Petushki Area, Volginsky, Vladimir Oblast 601125, Russia

**Keywords:** Immunology, Therapeutics, Cancer

## Abstract

In cancer immunotherapy, oncolytic viruses (OVs) offer an alternative approach to kill malignant cells by inducing cancer-specific host immune system activation. Given the ongoing need for safe and effective viral vectors for immunotherapy, non-zoonotic animal viruses have attracted significant interest. Here, we review the oncolytic myxoma virus (MYXV) and emphasize its emerging role in cancer immunotherapy. We critically discuss recent advances in cancer therapy using MYXV, primarily focused on engineered viral modifications. Furthermore, we briefly review alternative delivery strategies for MYXV and its combination with other anticancer agents. We also compare several key features of MYXV with those of other well-characterized OVs. In this study, we explore the key preclinical milestones that must be achieved for the MYXV OV to successfully transition to clinical trials. Preclinical studies have indicated the promising oncolytic potential and satisfactory safety profile of MYXV, thus paving the way for human clinical trials.

## Graphical abstract

**Figure:**
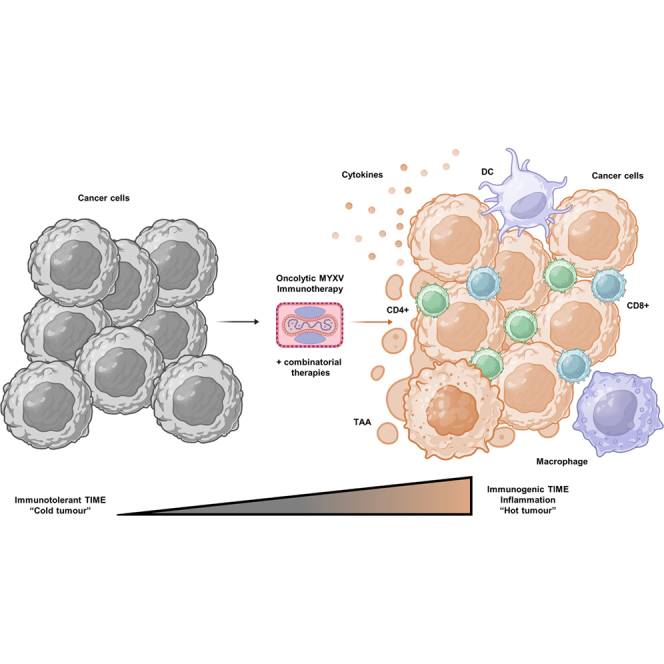

## Introduction

Oncolytic virus (OV) immunotherapy is an alternative therapeutic approach that uses wild-type or genetically engineered viruses to treat cancer. OVs can directly infect cancer cells, enhance innate and adaptive immune responses against tumor antigens, target cancer at metastatic sites, release tumor-associated antigens (TAAs), and activate and recruit immune cells into the tumor immune microenvironment (TIME).^1^^,^^2^^,^^3^ Cancer-specific virotherapy has been known for decades and still occupies a significant niche in the research and development pipeline for future cancer therapeutics. Since 2013, 289 trials have been conducted, of which 106 used OVs as monotherapy and 183 used OVs in combination with other therapies.^4^^,^^5^ OVs can directly lyse cancer cells but also induce immunogenic cell death (ICD) and promote the activation of specific anti-cancer immune responses by releasing TAAs.^4^ ICD is an initiating step in the cancer-immunity cycle that results in the TIME switching from an immunologically “cold” or deserted state to a “hot,” infiltrated-inflamed state. In this case, the tumor core and stroma are populated by tumor-antagonistic immune cells such as effector T cells, NK cells, dendritic cells, M1-polarized macrophages, and N1-polarised neutrophils.^6^ The ICD effect can be enhanced by the genetic modification of OVs, such as arming them with immunostimulatory molecules or deletion/knockout of anti-apoptotic genes. These unique properties of OVs make them a promising tool for the development of new combinatorial strategies to combat resistance in certain cancers or to circumvent poor drug penetration within the tumors. Several viruses have been extensively studied and tested for potential development as virotherapeutics. Only a few of them have been approved so far: H101 (Adenovirus), T-VEC (herpes simplex virus, HSV; Imlygic), ECHO-7 (Rigvir, registration withdrawn). The H101 is used in combination with chemotherapy in patients with nasopharyngeal carcinoma.^7^ The T-VEC is recommended for patients with stage IIIB to IV melanoma.^8^ The ECHO-7 was used for stage I and II melanoma.^9^ The Teserpaturev prescribed to treat glioblastoma following radiotherapy and temozolomide (TMZ)^10^ and nadofaragene firadenovec for BCG-unresponsive non-muscle invasive bladder cancer.^11^^,^^12^ The search for novel OVs can benefit translational cancer research and enhance the efficacy of complementary immunotherapy. Many viruses can be re-engineered to develop future virotherapeutics. However, not all are equally safe and effective. Patient safety and lack of virulence in humans are paramount in the design of OV vectors. Some animal viruses with a “clear” epidemiological history are increasingly being studied in the context of cancer immunotherapy. Among these viruses, myxoma virus (MYXV) is attracting particular attention due to its innate properties of being non-pathogenic to humans, its enormous genome capacity, and its ability to replicate selectively in human cancer cells.

The aim of this review is to summarize and update the current knowledge regarding MYXV as a potential vector for cancer immunotherapy. We discuss recent advances in preclinical studies involving MYXV and its engineered modifications. Furthermore, we critically assess combinatorial approaches that pair MYXV with other anti-cancer drugs.

We compare MYXV with other approved OVs and analyze the advantages and drawbacks of using MYXV for immunotherapies. Additionally, we highlight several key factors necessary for advancing MYXV into human clinical trials. We hypothesize that MYXV possesses several important oncolytic properties that make it a strong candidate for cancer virotherapy.

## Myxoma virus genetic and antigenic properties

**Figure 1:**
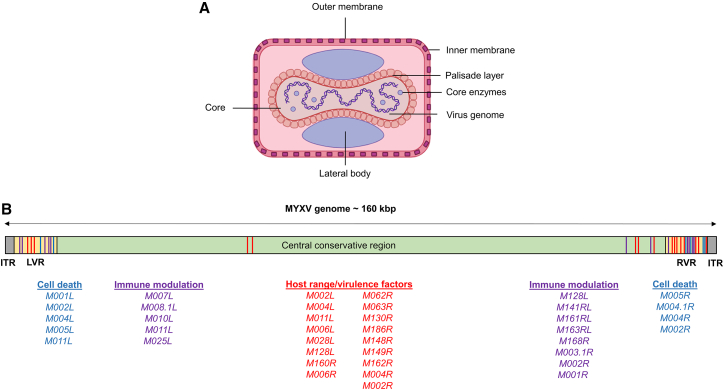
Schematic representation of MYXV structure and genome (A) MYXV virion consists of several distinct structures (core, inner and outer membrane, lateral body, DNA genome) and encapsulates numerous enzymes essential for virus replication and primary transcription. (B) MYXV double-stranded DNA genome (160 kbp in length) flanked by inverted terminal repeats (ITR) forming a harpin-loop structure. The 5’- (left variable region, LVR) and 3’- (right variable region, RVR) termini of the genome vary significantly between the MYXV strains. The LVR and RVR encode paralog genes involved in cell death (in blue), immune modulation (in purple), and host-range/virulence (red). The central part of the genome is relatively conserved and encodes viral proteins essential for replication and morphogenesis.

The MYXV is a member of the genus *Leporipoxvirus*, subfamily *Chordopoxvirinae*, family *Poxviridae* of DNA viruses.^3^^,^^13^ It is a large, brick-shaped virus that consists of a nucleocapsid core surrounded by a lipid membrane. The MYXV genome is a double-stranded DNA molecule of 161.8 kbp encoding 171 genes. It consists of a central region and two identical terminal genomic regions - inverted terminal repeats (ITRs).^13^ The central region is highly conserved among poxviruses and enriched with structural and housekeeping genes required for viral replication, transcription, translation, and virion assembly. The terminal regions of the MYXV genome consist of ITRs that form hairpin loops, creating a covalently closed molecule (Figure 1).

The genes encoded at these regions are unique to specific poxviruses as they encode host range or immunomodulatory proteins to subvert the host immune response and other antiviral pathways.^3^^,^^13^^,^^14^^,^^15^

MYXV has co-evolved with the indigenous *Sylvilagus californicus* or *Sylvilagus brasiliensis* species*,* causing a mild and non-lethal infection that causes only benign cutaneous lesions and is spread from host to host by biting arthropod vectors.^2^^,^^3^^,^^13^ Although transmitted by arthropod bites, MYXV does not replicate in arthropods, which act only as passive mechanical vehicles.^16^ However, MYXV causes a fatal disease called myxomatosis in European rabbits (*Oryctolagus cuniculus*), which was documented in the late 19th century when the virus was first transmitted by mosquito vectors from wild *Sylvilagus* rabbits to captive *Oryctolagus* rabbits imported to South America.^3^^,^^13^ In addition, many knockout constructs of MYXV, such as vMyx-*M135KO* and vMyx-*M063KO,* are non-pathogenic even in rabbits, while retaining their oncolytic properties against human cancer cells.^17^

**Figure 2:**
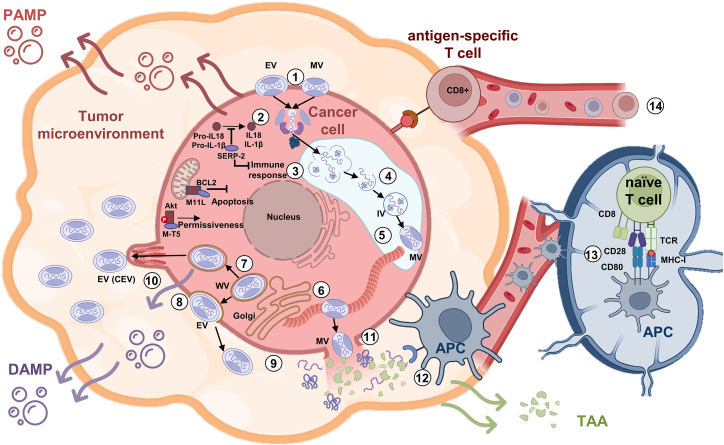
The mechanism of anti-cancer immune response activation by MYXV *The* MYXV replication cycle (1–11) begins with receptor-independent binding to the membrane and entry into the cancer cell via endocytosis. Subsequently, the virus core and lateral bodies are released into the cytoplasm. MYXV replication relies heavily on *M-T5*–Akt interactions. In addition, viral proteins such as *M11L* and *SERP-2* inhibit apoptosis and the innate immune response, respectively. The core is then transported through microtubules to the perinuclear region. Early gene expression occurs directly from the intact core and involves the core uncoating, inhibition of the host immune response, and transcription of intermediate genes. Furthermore, DNA replication also occurs subsequent to the release of the genome from the virus core. Subsequently, intermediate genes are expressed, modulating the transcription of late viral genes. The synthesis of virion proteins and the subsequent transcription of early genes are facilitated by late genes. The process of DNA binding to the membrane crescents gives rise to the formation of spherical structures known as immature virions. These immature virions then undergo a maturation process, during which they transform into mature virions (MVs). These mature virions are then transported through microtubules to the Golgi compartment. In the Golgi, the mature virion is enveloped by two additional membranes, resulting in the formation of a wrapped virion (WV). The vesicle is then transported to the cell membrane, where it is released by exocytosis. During this process, the WV loses two outer membranes, resulting in an exposed enveloped virion (EV). The virus can be found either bound to the membrane as a cell-associated enveloped virion (CEV) or in the medium as a free virus. Furthermore, CEV can be repelled from the membrane by actin outgrowths. The MYXV may exit a cancer cell through the process of lysis. This process has been demonstrated to elicit the release of tumor-associated antigens (TAAs) and damage- and pathogen-associated molecular patterns (DAMPs and PAMPs). These antigens are then captured by antigen-presenting cells (APCs), such as dendritic cells (12), and presented in the tumor-infiltrating lymph nodes as part of major histocompatibility complex (MHC) class I/II molecules to CD8^+^ and CD4^+^ T cells, respectively (13). This process has been demonstrated to initiate the priming and clonal expansion of these tumor antigen-specific CD8+ and CD4+ T cells, which are subsequently transported to the tumor microenvironment (14).

Despite its narrow natural host range, MYXV has been shown to infect several types of human cancer cells, largely due to: (1) the inability of most cancer cells to induce intrinsic antiviral responses and (2) the constitutive activation of intracellular pathways associated with cellular transformation, such as phosphorylation of Akt.^2^ MYXV has been shown to infect and kill over 70% of human cancer cell lines.^18^ The intracellular abnormalities in various cell signaling pathways in cancer cells, such as interferon (IFN)/tumor necrosis factor (TNF) responses or Akt activation, predispose myxoma to selective oncolysis.^15^ These findings encourage the investigation of MYXV oncolysis in other cancer models and possible anticancer applications, such as the use of MYXV in combination with other therapies. Combinatorial approaches may potential enhance MYXV immunotherapy efficacy.^19^ We summarize MYXV replication cycle and oncolytic mechanism in Figure 2, which depicts major changes in TIME and immune response.

## MYXV tropism

The cellular tropism of MYXV is very broad, since it can infect rabbit cells, certain primate cells, and human cancer cells.^20^ Currently, no specific receptors for MYXV binding are known. However, poxviruses known to bind to the molecules ubiquitously present on most animal cell surfaces, such as heparin sulfate, chondroitin sulfate, and glycosaminoglycans (GAGs), as well as extracellular matrix (ECM) protein laminin, and/or integrin β1. The most studied poxvirus (vaccinia virus) has an arsenal of proteins for cell attachment (i.e., A26, A27, D8, H3, and L1). It is shown that A27 and H3 are involved in GAG-dependent binding and bind to heparin sulfate, while D8 binds to chondroitin sulfate. It should be emphasized that mature virion (MV) and exposed enveloped virion (EV) have different viral surface epitopes and may use different attachment factors. The above-mentioned proteins are necessary for MV for cell attachment, while they have yet to be found for EV. Orthologues to vaccinia virus attachment proteins in MYXV are M115 (orthologue A27), M083 (orthologue D8) and M071 (orthologue H3). However, despite the similarity in surface protein structure, the binding mechanics of MYXV to cells can be different.^21^

Interestingly, MYXV does not bind to human CD34^+^ hematopoietic stem cells (SCs); however, binding and killing of malignant CD138+ myeloma cells have been demonstrated.^21^^,^^22^ The restriction of viral replication in cells is more likely determined by intracellular signaling pathways. The high tropism of MYXV for cancer cells is regulated by the defects in tumor suppressor pathways (e.g., mutations in p53, ataxia telangiectasia (ATM) and retinoblastoma protein (Rb), weak cellular IFN and TNF immune responses, increased protein expression of cancer cells, and upregulation of activated Akt.^23^ It has been demonstrated that the ability of MYXV to infect and kill human cancer cells is associated with basal levels of endogenous phosphorylated Akt, and that MYXV is able to switch tumor cells from a non-permissive to a permissive state by activating Akt through its viral host range ankyrin repeat protein, M-T5.^24^ The rabbit-restricted host range and high tropism to cancer cells make MYXV a very safe OV for *in vivo* studies in both immunocompetent and immunodeficient mouse models.^23^ In addition, recombinant strains of MYXV that are safe in rabbits but still active against cancer cells (*M135R* gene knockout) are available.^17^ MYXV replicates in cancer cells not only by disrupting signaling pathways, but also through its ability to modulate the immune response and ICD.

## MYXV immunomodulatory proteins

The MYXV genome encodes several genes capable of suppressing the immune response generated during virus replication. MYXV immunomodulatory proteins can be conditionally divided into 2 groups according to their localization: extracellular or membrane-associated viromimetics and intracellular virostats. The first group mimics host immune response factors and is subdivided into viroceptors (M-T1, M-T2, M-T7) and virokines (Serp-1, MGF) according to their mechanism of action.^25^ Viroceptors are homologous to cellular cytokine receptors, are either secreted from infected cells or are transmembrane viral proteins, and bind to immune ligands. Virokines mimic immune system inhibitors, growth factors, cytokines, and their agonists. Intracellular virostats inhibit recognition by the immune system or inhibit the induction of programmed cell death (PCD). This group includes viromitigators (Serp-2, M11L, M-T2, M-T4, M-T5) with anti-apoptotic or host range functions. In addition to viromitigators, there are intracellular virostealth strategies that involve the downregulation of cell surface markers (CD4, MHC I), thereby preventing the immune system from recognizing infected cells.^14^^,^^26^

A peculiar feature of poxviruses is the presence of homologs of host immune genes, which are necessary for the virus to evade an immune response and appear to be acquired during virus-host co-evolution.^27^ The immunomodulatory proteins of poxviruses can also be divided into several groups: intracellular (affecting signaling pathways related to DNA recognition, IFN, NF-kB), extracellular (cytokine and growth factor homologs, cytokine and chemokine receptor homologs), and modulators of cellular responses and complement activation. For more details on signaling pathways and their inhibition by poxviruses, see the review in.^27^ Here, we will only give examples of MYXV genes and their role in the induction of cell death, immunomodulation, and host-range pathways (Table 1).

**Table 1:** Myxoma virus proteins involved in cell death, immunomodulation, and host-range

Group	Modulator	Description	Predicted/Confirmed	Reference
Cell death regulator	*M002R/L (M-T2)*	MT-2 is able to inhibit apoptosis of myxoma-infected rabbit lymphocytes by binding to proapoptotic TNF-superfamily cell receptors.	confirmed	^13^^,^^17^^,^^25^
	*M004R/L (M-T4)*	MT-4 localizes to the ER, inhibits apoptosis, and is required for virus propagation in rabbit lymphocytes. The exact mechanism of apoptosis inhibition is not known. It is hypothesized that apoptosis may be inhibited by the ER itself through the regulation of gene expression in response to ER overload, or MT-4 may regulate the Bap31-complex with proapoptotic molecules such as Bcl-2/Bcl-XL, procaspase 8 in the ER.	confirmed	^13^^,^^17^^,^^28^^,^^29^
	*M005R/L (M-T5)*	MT-5 is required to prevent cell-cycle arrest in infected cells by manipulating Ub-proteosomal degradation of cell cycle control proteins, thus protecting the cell from regulated cell death pathways. MT-5 inhibits apoptosis in rabbit lymphocytes; its deletion inhibits viral and cellular protein synthesis.	confirmed	^13^^,^^17^^,^^30^
	*M011L (M11L)*	in the infected cell, M11L localizes predominantly to the outer mitochondrial membrane in a manner similar to cellular Bcl-2-like proteins. M11L inhibits apoptosis by preventing mitochondrial membrane permeabilization and cytochrome *c* release through interaction with proapoptotic Bak and Bcl2-family proteins, as well as inhibiting caspase-3 activation and poly(ADP-ribose) polymerase cleavage.	confirmed	^13^^,^^17^^,^^31^
	*M029L*	similar to the E3 protein of vaccinia virus, M029 inhibits the phosphorylation of PKR and eIF2α, ultimately reducing overall protein synthesis.	predicted	^13^^,^^15^^,^^32^
	*M143R*	M143R is a homolog of the RING-containing ubiquitin ligase p28 of orthopoxviruses and mediates the formation of polyubiquitin conjugates. It is hypothesized that M143R, like its homologous RING Finger Protein N1R of Shope Fibroma Virus, is capable of inhibiting apoptosis.	predicted	^13^^,^^33^^,^^34^
	*M148R*	contains up to 10 ankyrin repeats required for protein-protein interactions and 4 putative transmembrane regions. It is known that poxvirus proteins with ankyrin repeats can inhibit apoptosis.	predicted	^13^^,^^17^
	*M149R*	contains up to 9 ankyrin repeats required for protein-protein interactions. It is known that poxvirus proteins with ankyrin repeats can inhibit apoptosis.	predicted	^13^^,^^17^
	*M150R (MNF)*	contains 9 ankyrin repeats required for protein-protein interactions, with 8 ankyrin repeats having a close similarity with the nuclear localization signal-containing ANK of I-κBα. It is known that poxvirus proteins with ankyrin repeats can inhibit apoptosis.	predicted	^13^^,^^17^^,^^35^
	*M156R*	M156R structurally mimics the Eukaryotic Translation Initiation Factor eIF2α, whose phosphorylation is required for ER stress and immunogenic cell death.	predicted	^13^^,^^17^^,^^36^
	*M151R (SERP-2)*	SERP-2 is a member of the serine proteinase inhibitor (serpins) superfamily. It is an anti-apoptotic protein due to the inhibition of caspases.	confirmed	^25^^,^^37^^,^^38^
Immuno modulation	*M001R/L (M-T1)*	an inhibitory glycoprotein capable of high binding to CC-chemokines that normally activate and mobilize immune cells (monocytes/macrophages, T lymphocytes, NK cells, dendritic cells) to sites of inflammation.	confirmed	^13^^,^^25^
	*M002R/L (M-T2)*	soluble tumor necrosis factor receptor (TNFR), which binds to TNF ligands prior to their interaction with cell surface receptors, thus preventing the antiviral response, which is expressed by TNF-induced cytolysis of the infected cell, apoptosis, and inhibition of viral replication.	confirmed	^13^^,^^25^
	*M007R/L (M-T7)*	soluble IFN-γ receptor homolog, a lymphocyte-derived cytokine, is able to bind to rabbit IFN-γ with high affinity and inhibit IFN-γ binding to cellular receptors, thereby interfering with antiviral immunity, including inhibiting leukocyte infiltration at the site of infection and binding to CXC, CC, and C-subfamily chemokines through their heparin-binding domain. This viral protein is secreted in the highest amounts, is synthesized early in infection, and is stable long after infection. M-T7 represents the major virulence factor.	confirmed	^13^^,^^25^
	*M008.1R/L (SERP-1)*	SERP-1 belongs to a family of serine proteinase inhibitors (serpins) that regulate the modulation of inflammatory responses. SERP-1 restrains the inflammatory response *in vivo*.	confirmed	^13^^,^^25^
	*M010L (MGF)*	MGF (myxoma growth factor) is a homolog of epidermal growth factor (EGF) and is able to bind to the EGF receptor. MGF stimulates cellular biosynthesis, thereby creating a more favorable environment for virus replication.	confirmed	^13^^,^^25^^,^^39^
	*M029L*	M029L is a member of the poxvirus E3 family of dsRNA-binding proteins that antagonize the cellular interferon signaling pathways. M029L mobilizes proviral RHA/DHX9 to stimulate viral replication in myeloid cells.	confirmed	^13^^,^^15^
	*M104L*	M104L has IL-8 receptor-like fragment.	predicted	^13^^,^^15^^,^^17^
	*M121R*	M121R exhibits homology to human NKG2, a member of the natural killer cell (NKC) receptor family, which binds to class I MHC on target cells and inhibits NKC-mediated cytotoxicity, which is normally responsible for immune surveillance to eliminate cells with decreased class I MHC expression levels.	predicted	^13^^,^^17^
	*M122R*	M122R exhibits homology to murine Ly-49, a member of the natural killer cell (NKC) receptor family, which binds to class I MHC on target cells and inhibits NKC-mediated cytotoxicity, which is normally responsible for immune surveillance to eliminate cells with decreased class I MHC expression levels.	predicted	^13^^,^^17^
	*M128L*	M128L has a homology to the cellular CD47 protein (integrin-associated protein, or IAP) and inhibits the activation of myeloid-lineage cells within the host.	confirmed	^13^^,^^17^^,^^40^^,^^41^
	*M135R*	M135R encodes a cell surface protein that is predicted to mimic the host alpha/beta interferon receptor (IFN-α/β-R) and thus prevent IFN-α/β from triggering a host antiviral response. However, it was demonstrated experimentally that the target immune ligand for M135R is not from the predicted type I interferon family and remains to be identified.	predicted	^13^^,^^42^
	*M141R*	M141R has significant amino acid similarity to the family of cellular CD200 (OX-2) proteins involved in the regulation of myeloid cell activation. M141R inhibits macrophage activation in infected lesions and draining lymph nodes by rapidly downregulating NF-κB responses to viral infection, and inhibits the activation of circulating T lymphocytes during infection.	confirmed	^13^^,^^43^^,^^44^
	*M144R*	M144R has homology to vaccinia virus genes C3L (vaccina complement control protein, VCP), which blocks the activation of the complement system, and B5R, which, as a C3L, exhibits a similar relatedness to the RCA (regulator of complement activation) superfamily.	predicted	^13^^,^^15^^,^^45^
	*M146R*	M146R has homology to vaccinia virus protein N1L, an inhibitor of pro-apoptotic Bcl-2 family.	predicted	^13^^,^^46^
	*M148R*	contains up to 10 ankyrin repeats required for protein-protein interactions and 4 putative transmembrane regions. It is known that poxvirus proteins with ankyrin repeats can influence NF-κB activity.	predicted	^13^^,^^17^
	*M149R*	contains up to 9 ankyrin repeats required for protein-protein interactions. It is known that poxvirus proteins with ankyrin repeats can influence NF-κB activity.	predicted	^13^^,^^17^^,^^47^
	*M150R (MNF)*	myxoma nuclear factor (MNF) seems to interfere with NF-κB (NF-κB) pathways, leading to the inhibition of the inflammatory response.	predicted	^13^^,^^17^^,^^48^
	*M151R (SERP-2)*	SERP-2 is a member of the serine proteinase inhibitor (serpins) superfamily. Inhibition of ICE/caspase 1 prevents the maturation of the inflammatory cytokine IL-1β.	confirmed	^13^^,^^25^
	*M152R*	M152R encodes a serine proteinase inhibitor (Serp3).	predicted	^13^^,^^15^^,^^49^
	*M153R (LAP)*	M153R, a viral E3 ubiquitin ligase, downregulates MHC class I molecules, Fas, and the T cell coreceptor CD4 and inhibits cytolysis by antigen-specific CTLs. M153R enhances NK-mediated anti-glioma activity. M153R has a PHD/LAP zinc finger domain.	confirmed	^50^^,^^51^^,^^52^^,^^53^
	*M156R*	M156R structurally mimics the eukaryotic translation initiation factor eIF2α and is an efficient substrate for phosphorylation by PKR and can compete with eIF2α. The gene also has homologous sites with immunomodulatory proteins of poxviruses (C8L swinepox virus and K3L vaccinia virus) that act as inhibitors of the interferon-inducible dsRNA-dependent protein kinase, PKR. In the antiviral response, PKR is induced by interferon and activated by dsRNA and then phosphorylates eIF2α, resulting in translational inhibition that prevents synthesis of viral proteins.	confirmed	^36^
	*M011L (M11L)*	M011L knockout results in more active leukocyte infiltration and an effective immune response in rabbits.	confirmed	^25^
	*M002 R/L (M-T2)*	M-T2 is a soluble tumor necrosis factor receptor (TNFR). M-T2, in conjunction with M-T5 and M11L, enables replication in infected lymphocytes. Disruption of both copies of the M-T2 gene in myxoma virus results in a significantly attenuated disease phenotype, with primary and secondary lesions smaller than those in control rabbits infected with wild-type myxoma virus.	confirmed	^21^^,^^54^
	*M004 R/L (M-T4)*	the M-T4 knockout virus causes only some clinical signs of myxomatosis, and rabbits recover completely from infection. The mutant virus is unable to spread to secondary foci of infection because it is unable to replicate in the leukocytes that normally spread it.	confirmed	^13^^,^^28^
Host range/virulence factors	*M005 R/L (M-T5)*	M-T5 is a critical host range factor that inhibits apoptosis of infected lymphocytes. Its deletion results in non-lethal infection of the rabbit, and virus spread is limited to the site of introduction by a rapid antiviral immune response.	confirmed	^17^
	*M011L (M11L)*	M11L is an important virulence factor. When M011L is knocked out, the virus becomes attenuated and does not cause rabbit death, demonstrating more active leukocyte infiltration and an effective immune response.	confirmed	^25^
	*M029L*	when M029L is knocked out, the virus is attenuated and does not cause myxomatosis. M029L is required for virus replication in all mammalian cells by counteracting PKR-mediated antiviral functions.	confirmed	^32^^,^^15^
	*M062R*	M062 is a member of the conserved C7L superfamily of poxvirus proteins and is essential for MYXV infection. M062 inhibits sterile α motif domain-containing 9 (SAMD9), a protein constitutively expressed in mammalian cells and involved in the antiviral immune response. Infection by MYXV with the M062R gene knockout was completely asymptomatic.	confirmed	^55^^,^^56^
	*M063R*	it shares homology with vaccinia virus C7L. M063 can bind to M062 to form a complex that antagonizes the cellular antiviral factor SAMD9. When M063R is knocked out, the virus does not cause signs of myxomatosis in rabbits, but functions as a non-replicating vaccine and provides protection against further infection with the wild type	confirmed	^57^
	*M128L*	M128L is necessary for the production of a lethal infection in susceptible rabbits, while it is fully dispensable for virus replication *in vitro*.	predicted	^40^^,^^41^
	*M130R*	the loss of M130R expression allows the rabbit host immune system to effectively respond to and control the lethal effects of MV. M130R is required for full-fledged myxomatosis in European rabbits.	predicted	^58^
	*M135R*	when the M135R gene was deleted, MYXV was attenuated *in vivo,* and there was no development of myxomatosis.	predicted	^42^
	*M148R*	contains up to 10 ankyrin repeats required for protein-protein interactions and 4 putative transmembrane regions. The gene deletion does not affect cell tropism or host range, but induces a delay in the onset of blepharitis and respiratory infections, an increase in survival time, and a dramatic decrease in mortality rate. In MYXV-infected rabbits with the deletion of both M148R and M149R genes, no signs of respiratory infection are observed, and a complete recovery is subsequently observed	predicted	^42^
	*M149R*	contains up to 9 ankyrin repeats required for protein-protein interactions. Deletion of the gene does not affect cell tropism or host range, but induces a delay in the onset of blepharitis and respiratory infections, an increase in survival time, and a dramatic decrease in mortality rate. In MYXV-infected rabbits with the deletion of both. M148R and M149R genes, no signs of respiratory infection are observed and a complete recovery is subsequently observed.	confirmed	^48^
	*M150R*	M150R encodes myxoma nuclear factor (MNF), which is critical for productive viral infection in rabbits, since its deletion generates an almost apathogenic virus that still replicates in cells.	predicted	^35^
	*M152R (SERP-3)*	M152R encodes Serp-3, a member of the serine proteinase inhibitor (serpins) superfamily. Its deletion results in attenuated European rabbit disease and the inability of the virus to cause myxomatous lesions of draining lymph nodes.	confirmed	^17^
	*M156R*	M156R is a homolog of K3L of vaccinia virus, which acts as a pseudo-substrate for PKR and eIF2α. Inhibits protein synthesis in the cell.	confirmed	^17^^,^^36^

### MYXV protein regulators of cell death

PCD of infected cells prevents the virus from spreading within the host. PCD may also be a consequence of the cellular antiviral IFN response. Pattern recognition receptors (PRRs) regulate the production of IFNs and the subsequent activation of IFN-stimulated genes (ISGs). ISGs encode proteins with antiviral activity and diverse functions, including a group of PCD inducers such as caspases. Inflammatory caspases (caspase-1, 4, 5, 11, and 12) are involved in inflammasome activation and pyroptosis, whereas apoptotic caspases (caspase-2, -3, -6, -7, -8, -9, and -10) are widely regarded as non-inflammatory.^59^^,^^60^ Many reviews have focused on PCD and the effects of poxviruses on it.^59^^,^^60^^,^^61^^,^^62^ Table 1 summarizes the MYXV proteins that modulate PCD with a description of their functions.

## Armoring strategies for oncolytic MYXV

The MYXV can be genetically and biochemically modified to improve its safety profile (e.g., enhanced tumor targeting, miRNA, and cancer cell-specific replication), anti-cancer activity, and modulate TIME (e.g., anti-angiogenesis, metabolic reprogramming, and ECM barrier penetration).^17^^,^^58^^,^^63^^,^^64^^,^^65^^,^^66^ We summarized the MYXV modifications (Table S1) and provided some examples with the mechanism of action in this section.

### The vMyx-M135KO

This recombinant vMyx-*M135KO* shows attenuated disease phenotype in European rabbits and has enhanced oncolytic activity in cancer cells; it has been proposed as a leading candidate for phase I clinical trials. It has been tested in small-cell lung cancer (SCLC).^67^^,^^68^ The recombinant vMyx-*M135KO* virus had a high safety profile tested in normal (non-malignant) mammalian cells, high cytotoxicity against human and mouse SCLC tumor lines, as well as freshly obtained human SCLC samples and patient-derived xenograft (PDX) models. Treatment with this recombinant virus prolongs overall survival (OS) when used intratumorally in 2 doses 48 h apart, with strong induction of host immune cell defense. Extensive necrosis was observed following intratumoral injections, which may be associated with host immune cell infiltration. The MYXV was cleared rapidly, which is associated with sustained immune cell infiltration, suggesting that MYXV-infected SCLC undergoes immunologic cell death in immunocompetent models. It enhanced immunostimulatory response, demonstrated by the increase of CD45^+^ markers.^67^^,^^68^

### The vMyx-M011LKO

Knockout of the *M011L* gene in MYXV induces caspase-3/7-dependent apoptosis in brain tumor initiation cells (BTICs) isolated from glioma surgical specimens. Treatment with vMyx-*M011LKO* did not prolong survival in immunocompromised mice, but it increased survival to 30% in immunocompetent mice, suggesting that innate immune responses are important for successful replication of the virus. It also increased CD3^+^ cell infiltration, but did not generate anti-tumor immunity.^69^

### The vMyxΔSERP-2

This recombinant MYXV has a deletion of a viral anti-apoptotic protein, Serp-2, which attenuates pathogenesis in rabbits and enhances oncolytic effects on cancer cells. Clinical signs (lethargy, hunched posture, difficulty moving, significant weight loss) were reduced in mice with alveolar rhabdomyosarcoma, but there was no significant improvement in survival.^70^

### The MYXV-mIL15

This recombinant virus variant encodes murine interleukin-15 (mIL-15) that enhances the proliferation of natural killer (NK) cells, CD8^+^ T cells, and memory T cells, facilitating the recruitment of cell-mediated immune response to the tumor bed. Using MYXV-mIL15, melanoma was treated in immunocompetent mice, but without significant changes in survival and metastases. Interestingly, metastases in the lymph nodes and lung were more common in mice treated with the recombinant viruses. Virus treatment induced neutrophilic inflammation at the injection site. The MYXV-*mIL15* increased CD3^+^ T lymphocytes, but this did not correlate with prolonged survival or a reduction in the number of metastatic lesions.^71^

In order to increase the stability of the IL15 molecule and enhance its bioavailability, a fusion construct (***α*** subunit of the IL15 receptor (IL15R***α***) and mIL-15) was designed to generate a recombinant virus (vMyx-IL15Ra-tdTr). Melanoma treated with fusion protein expressing MYXV showed attenuated tumor growth and prolonged survival, as well as increased infiltration of NK cells and CD8^+^ T cells.^72^ In a glioma model, the use of vMyx-IL15R***α***-IL15 promoted tumor infiltration of Ly-49G2+ NK cells and CD9+T cells. Although this immunotherapy approach was safer than vvDD-IL15R***α***-IL15 in mice, it has a toxic effect when injected intracerebroventricularly. Nevertheless, the survival benefit was modest, likely because the CD8^+^ T cells lacked tumor specificity and the TIME in the brain is immunosuppressive, limiting their effectiveness.^73^

*The vMyx-PD1/IL12* is a recombinant MYXV that expresses both a soluble PD1 inhibitor and interleukin-12 (vPD1/IL12). The vMyx-*PD1/IL12* has demonstrated good activity against various tumor models.^74^ Valenzuela-Cardenas et al.^74^ tested this recombinant virus on Lewis lung carcinoma, melanoma, colon adenocarcinoma, and ovarian cancer and were able to induce tumor regression. In lung and ovarian cancer models, complete phenotypic elimination of tumors and long-term survival was registered. All animals rechallenged with tumor cells were able to reject them. In melanoma and colon adenocarcinoma, OS was improved, but animals were not cured. The vMyx-*PD1/IL12* can induce fully curative responses depending on functional T cell immunity, which may be compromised in unresponsive models. This recombinant virus induced high levels of inflammatory cytokine expression (increased expression of IFN-γ and TNF). In the follow-up study, its oncolytic potential was tested in triple-negative breast cancer.^75^ The vMyx-*PD1/IL12* delayed tumor growth and improved OS, including in the metastatic 4T1 *in vivo* model. Intriguingly, vMyx-PD1/IL12 also reduced metastatic tumor burden in the lungs.^75^

### The vMyx-CD47/IFNγ

The recombinant virus vMyx-CD47/IFNγ contains two additional genes integrated into its genome: the murine integrin-associated protein (mCD47) and murine interferon-γ (mIFN- γ). The mCD47 functions to limit the rapid clearance of MYXV-infected cells by phagocytes, while mIFN-γ promotes the adaptive immune response by activating T cells and enhancing antigen presentation. Mechanistically, overexpression of CD47 delays the clearance of MYXV, allowing IFN-γ to accumulate at the tumor site. In melanoma models, this dual-armed recombinant MYXV demonstrated superior anti-cancer efficacy by inhibiting tumor growth compared to the wild-type virus and single-armed versions expressing either CD47 or IFN- γ alone. Since the replication rates of the viruses were similar, the enhanced efficacy of vMyx-*CD47/IFNγ* is likely due to its ability to activate anticancer immunity via modulation of the TIME. Increased infiltration and activation of CD3^+^ and CD8^+^ T cells supports this finding. Additionally, elevated co-expression of IFN-γ with CD8^+^ T cells and granzyme B+ CD8^+^ T cells indicates an anti-tumor immune response activation.^76^

*The vMyx-mLIGHT-Fluc/tdTr* is another example of recombinant MYXV that expresses the *LIGHT* gene, which encodes member 14 of the murine tumor necrosis factor ligand superfamily (TNFSF14). This expression enhances the recruitment of T lymphocytes and NK cells to the tumor site and activates the immune response, promoting tumor-specific memory T cell stimulation.^77^^,^^78^^,^^79^ It has pro-apoptotic effects on some tumor cells through the lymphotoxin-beta receptor and the release of tumor.^79^ Histopathological evaluation of H&E-stained tissues revealed lymphocytic infiltrates upon the injection of *LIGHT*-armed MYXV, leading to the modulation of the TIME in pancreatic ductal adenocarcinoma (PDAC) tissues. When used as monotherapy, it prolongs survival and reduces tumor burden, but its efficacy is limited and must be combined with other therapies or delivery methods.^77^^,^^78^ In a lung metastatic osteosarcoma model, this recombinant virus was shown to be effective in reducing tumor burden when shielded with peripheral blood mononuclear cells (PBMCs).^77^

*The* vMyx-*hTNF* expressing human TNF was designed and used as a monotherapy against drug-resistant Vk∗MYC murine myeloma (MM). The authors used an autologous bone marrow *ex vivo* loaded with unarmed-MYXV or TNF-armed MYXV to improve virus delivery and avoid early anti-viral immune response. The data show a delay in the onset of MM disease *in vivo* and improve survival rates in mice pre-seeded with BOR-resistant murine Vk12598 myeloma cells. However, preclinical studies also report that no statistically significant changes were found between the groups treated with vMyx-*hTNF* and unarmed virus. According to the study, the method of viral delivery plays a significant role in the effectiveness of the therapy when autologous bone marrow was loaded *ex vivo* with either unarmed-MYXV or NF-armed MYXV, the survival rate of mice reached approximately 50%, compared to 10–20% for systemic administration. Nevertheless, it is worth noting that this study is limited to mouse survival as an indicator of therapy efficacy, without examining tumor burden using, for example, flow cytometry to count CD138+ cells, which is crucial for understanding the progression of the disease. Moreover the authors measure the circulating M-Spike protein level to determine tumor burden in groups of mice that additionally received immunotherapy with α-PD-1 and the mimetic compound Smac LCL161, an antagonist of the cellular apoptosis inhibitor cIAP-1 and -2. Combinatorial immunotherapy may outperform monotherapy using oncolytic MYXV, the individual contribution of the virus to the measured effect is difficult to determine.^68^^,^^79^

MYXV encoding fusogenic proteins. Viral fusogenic proteins are transmembrane glycoproteins found in enveloped viruses that mediate the fusion between the viral envelope and the host cell membrane. Membrane fusion of infected cells forms a multinucleated cell body called a “syncytia,” which facilitates viral spread and improves their therapeutic efficacy. Burton et al.^66^ created four different recombinant MYXV containing F proteins from different virus origins: vMyx-NDV-F (Newcastle Disease Virus),vMyx-RSV-F (respiratory syncytial virus), vMyx-NV-F (Nipah Virus), and vMyx-BPV-F (Bovine Parainfluenza Virus) and tested them in a Lewis lung carcinoma model. All of these viruses, rather than reducing tumor burden and prolonging survival, actually increased tumor growth rates, overall tumor burden, and decreased survival compared to the control virus. The efficacy and viral titer of MYXV were notably reduced, especially in highly fusogenic constructs such as vNDV and vRSV, likely due to rapid cell lysis caused by syncytia formation. The authors highlight a critical concern regarding the negative impact of fusogenic proteins when used for tumor treatment in *in vivo* models.

Readers seeking further information on biochemical approaches, with a focus on virus engineering and advancements in immunotherapy, are referred to our previous review.^4^ This review discusses OV transduction targeting methods (e.g., xenotype switching, pseudotyping, and cell receptor targeting) and non-transduction modifications (e.g., microRNA, optogenetics, transcriptional targeting, and tissue-specific promoters).

## Combinatorial approaches in anti-cancer therapy using MYXV

Cancer cells possess certain characteristics that make them difficult to eliminate. For example, cancer cells « shield » themselves from the immune system through progressive immune editing.^80^ Factors such as immunosuppressive cytokine patterns, reduced activity of immune effector cells, impaired immune cell migration toward tumors, reduced antigen presentation, and changes in major histocompatibility complex (MHC) expression facilitate immune evasion in cancer and allow the disease to spread.^80^ Monotherapies are often insufficient as they can lead to drug resistance due to the heterogeneity and complex genetic mutation burden in tumors and complex TME. OVs are flexible agents that can be used in addition to other types of therapies, such as chemotherapy, immune checkpoint inhibitors (ICIs), targeted drugs, and adoptive T cell therapies.^81^ Here, we provide some examples of combinatorial approaches that have been used with MYXV.

### Chemotherapy

*Cisplatin* is a metal-based alkylating agent that binds to genomic or mitochondrial DNA to form lesions, blocks the production of DNA, mRNA, and proteins, arrests DNA replication, and inhibits cell division.^82^^,^^83^ It is a first-line drug for several cancers such as testicular,^84^ ovarian,^82^ bladder,^85^ lung,^86^ cervical,^87^ gastric,^88^ and others. However, cisplatin has many drawbacks, including resistance and multiple side effects such as nephrotoxicity, ototoxicity, hepatotoxicity, and gastrointestinal neurotoxicity.^83^ However, it also affects the host immune response by reducing regulatory T cells while enhancing antigen-specific CD8^+^ T cell activities.^89^ The combination of the MYXV *M135KO* with low-dose cisplatin for the treatment of SCLC was effective, as the OS was improved compared to the monotherapy with cisplatin or PBS alone. Nevertheless, increased survival was also documented with the viral monotherapy.^67^ In a disseminated. The combination of vMyx-*M062RKO* or vMyx-WT with cisplatin was tested in ovarian cancer associated with an immunosuppressive tumor microenvironment model (SKOV3 cells). The study shows that OS was improved. Particularly, the ovarian cancer treatment regimen, in which treatment with replication-competent MYXV precedes the addition of cisplatin, allows for the effective activation of the two drugs: MYXV changes the tumor microenvironment, increasing the sensitivity of cancer cells to the effects of cisplatin. In contrast, if the drugs were used in the opposite order, cisplatin would inhibit the replication and antitumor effects of MYXV. To achieve the most effective synergistic effect of MYXV with chemotherapy, it is necessary to consider the sequence of drug administration and to further investigate the mechanisms of action of both drugs, not only on cancer cells but also on each other.

TMZ is a derivative of dacarbazine with alkylating properties, it has the ability to cross the blood-brain barrier (BBB), it induces DNA methylation and alkylation, damaging it and leading to apoptosis.^90^ It has some side effects that are well tolerated by patients, such as fatigue, nausea, vomiting, myelosuppression, and hematological complications.^91^ Resistance is also quite a problem, which can be avoided by using it in combination with TMZ-enhancing agents, targeted therapies, and immunotherapies.^91^ The recombinant MYXV (vMyx), *vMyx-M011LKO*, in combination with TMZ, produced durable responses in immunocompetent animals. The vMyx-*M011LKO* alone had no effect on caspase 3 activation, but in combination with TMZ, it increased the number of cleaved caspase 3-positive cells.^69^

### Gemcitabine (GEM)

This pyrimidine nucleoside antimetabolite is a first-line drug used to treat PDAC. It works by incorporating into DNA, inhibiting strand elongation, inducing reactive oxygen species (ROS), blocking the cell cycle, and inducing apoptosis.^92^^,^^93^ Gemcitabine reduces Treg activity, increases NK cell activity and IL-12 production, and alters the ratio of cytotoxic T lymphocytes (CTL) to Treg in the TME. In the study by Jazowiecka-Rakus et al.,^78^ when GEM was used as monotherapy or in combination with vMyx-*mLIGHT*-Fluc/tdTr to treat PDAC, it had a detrimental effect on the survival of the animals, even though it was used at a concentration equal to half the maximum tolerated dose. Apparently, the sequence of treatment in combinatorial therapies may significantly affect the efficacy of immunotherapy. According to Wennier S.T. et al.,^94^ the best way to administer both therapies is to give the MYXV first and then the gemcitabine, stating that this approach resulted in 100% long-term survivors in immunocompetent mice. They have proposed that myxoma infection triggers an adaptive immune response to both viral and tumor antigens, and that these antitumor immune responses sensitize the cancer cells to the cytotoxic effect of gemcitabine.

The combination of MYXV and gemcitabin for pancreatic cancer therapy significantly prolonged survival and reduced tumor burden compared to either agent alone. The proposed mechanism is based on the MYXV ability to interfere with DNA repair pathways and cell cycle regulation, which increases gemcitabine-induced DNA damage. MYXV also reduces tumor cell resistance by sensitizing to nucleoside analog incorporation.^94^ In addition, MYXV replication enhances apoptosis signaling (caspase-3, PARP cleavage). Moreover, immune system stimulation (recruitment of T cells) was observed to be synergized with drug-mediated cytotoxicity.

*Cyclophosphamide (Cy)* is an alkylating agent that is not cell cycle phase specific. It potentiates T cell redirected therapy and other adaptive immune responses against tumors through the diminution of Treg and restoration of T and NK cell functions.^68^^,^^95^ The use of combinatorial therapy using Cy and vMyx-*M135KO* or vMyx-*hTNF* « shielded » by bone marrow cells was tested for treating BOR-resistant multiple myeloma (MM). The data showed that either shielded or “naked” MYXV virus could delay the onset of established MM disease by inducing a moderate improvement in survival rate. However, the effect was not significantly different from the mice treated with Cy only. This could have happened because it is known that Cy stimulates antiviral immune defenses. Alternatively, Cy may compromise the spread of the virus in the tumor milieu, or the combination may be toxic.^68^

### Adoptive T cell therapy

Adoptive T cell therapy is a type of immunotherapy in which *ex vivo* engineered T cells are infused to induce an immune-mediated anti-tumor response.^96^ There are two main widely used approaches: (1) use of naturally occurring tumor-specific T cells extracted from existing tumor masses (tumor-infiltrating lymphocytes, or TILs) and (2) genetically modified blood-derived T cells engineered to specifically recognize tumor cells (CAR T cells). While these therapies can be effective, tumors can sometimes evade them, such as by developing antigen-loss variants, which allow them to escape detection by antigen-specific T cells.^97^ The combination of this therapy with OV can enhance therapy through oncolysis, which would release tumor antigens that stimulate T cell responses and recruit additional immune cells, cytokine production, and expression of therapeutic genes.^97^ The combination of CD8^+^ 2C T cells with vMyx doubled survival in mice with melanoma brain tumors that lack endogenous T and B cells.^97^ The combination of adoptive GARC-1 tumor-specific CD8+ T cell transfer (boosted by TriVax), rapamycin with celecoxib, and vMyx-*IL15R****α****IL15* cured 83% of mice with glioma, with reduced toxicity.^73^ Nevertheless, the combination of MYXV and CAR T cell therapy for blood cancer treatment seems problematic and challenging due to the low anticancer effect after virus systemic administration.

### Immune checkpoint inhibitors (ICIs)

*Anti-programmed cell death ligand-1 (PD-L1) antibodies (atezolizumab, durvalumab, avelumab).* Anti-PD-L1 monoclonal antibodies, including atezolizumab (Tecentriq), durvalumab (Imfinzi), and avelumab (Bavencio), are FDA-approved ICIs that block PD-L1 binding to PD-1 on T cells, thereby boosting antitumor immunity. These agents have achieved durable remissions, particularly in PD-L1-high cancers such as non-small cell lung cancer (NSCLC; e.g., atezolizumab + bevacizumab in metastatic cases), urothelial carcinoma, and Merkel cell carcinoma. However, many tumors are considered immunologically “cold” - they have a low mutational burden and limited neoantigen expression. As a result, these tumors exhibit poor infiltration by cytotoxic T cells and are characterized by a highly immunosuppressive TIME, making them less responsive to immunotherapy. Infection of tumor-bearing mice with MYXV can increase PD-L1 expression at the tumor site. In the study by Woo et al.,^76^ melanoma was treated by combining vMyx-CD47/IFN**γ** with ***α***PD-L1. Inhibition of tumor growth with the combination of vMyx-CD47 with ***α***PD-L1 was stronger than after monotherapy. The combination treatment promoted the infiltration of immune cells (CD8^+^ T cells), increased the anti-cancer activity of the infiltrating immune cells, and prolonged survival. Interestingly, a reduction of the Treg population after MYXV injection was observed. This might be an important marker since Tregs suppress excessive immune responses and can inhibit the therapeutic effects of ICIs. Thus, the combination with a virus can potentiate the activity of ICIs in solid tumors.^76^ In combination with vMyx-hTNF or vMyx-m*LIGHT*, established tumors of metastatic osteosarcoma in the lung can be treated, unlike monotherapies (virotherapy or immunotherapy), which lose efficacy in later stages of the disease.^79^

### Anti-CTLA-4 antibodies (ipilimumab (Yervoy), tremelimumab)

The combination of PBMC-loaded vMyx-*hTNF* with checkpoint inhibitors (anti-PD-1, anti-PD-L1, or anti-CTLA-4) led to complete responses in a substantial fraction of mice, with some cohorts showing near-100% survival to the end of follow-up (e.g., 80–100% vs. 0–20% in monotherapy or control groups at ∼70 days. Flow cytometry of lung and/or systemic immune compartments showed increased infiltration/activation of effector immune cells (e.g., CD8^+^ T cells and other cytotoxic subsets) in vMyx-*hTNF*-treated mice compared with controls. The vMyx-*hTNF* induced higher levels of several cytokines and chemokines than unarmed MYXV in serum or *ex vivo* assays, including key mediators of innate and adaptive immunity (e.g., increased TNF-α itself plus elevated IFNs and T-cell-associated cytokines; many increased several-fold over baseline).^98^

### Other anti-cancer drugs and MYXV

#### Rapamycin

Rapamycin is a macrocyclic lactone that increases endogenous *p*-Akt and can render the type II cancer cell lines more permissive to MYXV infection, acting as an immunosuppressant by altering the host's innate or adaptive cellular immunity that facilitates infection.^99^ It has been shown to prolong survival in combination with WT MYXV for the treatment of immunocompetent models of malignant glioma, and also to reduce the recruitment of CD68^+^ and CD163+ cells.^20^ In immunosuppressed mouse models of medulloblastoma, this combination reduced spinal cord and ventricular metastases.^100^ However, this approach was not effective in reducing tumor area or prolonging host survival of glioma xenografts (GBC-SD).^99^

*Selinexor* (selective nuclear export inhibitor). Exportin-1 (XPO1) is an export receptor for several proteins, both oncogenes and oncosuppressors, and has been found to be overexpressed in many cancers. The selective inhibitor of nuclear export (selinexor) targets XPO1, resulting in selective apoptosis of cancer cells.^101^ Inhibition of XPO1 or CRM1 (chromosome region maintenance 1) with selinexor increases MYXV gene expression, replication, and progeny formation in cell lines resistant to myxoma replication by reducing the appearance of cytoplasmic antiviral granules composed of the RNA helicase DHX9.^102^ Potentially, this stress response created a more permissive environment for MYXV replication. In models of colorectal adenocarcinoma and epithelioid carcinoma, the combination of selinexor with vMyx-Fluc reduced tumor burden and prolonged survival.^102^ The data indicated a stronger immune response and tumor reduction in combination (MYXV and Selinexor) than either agent alone.

*Hyaluronan* (HA) is a large GAG that is a component of the ECM and contributes to cell proliferation and migration. Its binding to CD44 isoforms activates PI3K/Akt/mTOR, which increases *p*-AKT, and induces the expression of metalloproteinase-9 (MMP-9, gelatinase B), which degrades collagens, which are components of the ECM and present a barrier to therapeutic molecules in tissues, and may interfere with the efficacy of virotherapy. In combination with MYXV for the treatment of GBC, it increased oncolytic efficacy compared to MYXV+Rampamycin in immunodeficient mice, prolonged survival, and reduced tumor size.^99^

*Celecoxib* is a cyclooxygenase-2 (COX-2) inhibitor that targets prostaglandin E2. The product of COX-2 activity causes dendritic cells to induce Tregs, maintains myeloid-derived suppressor cells (MDSCs), and inhibits the ability of antigen-specific T cells to kill tumor targets. Tumor cells induce the expression of COX-2 in infiltrating myeloid cells.^73^ It can be used as an enhancer of immunotherapies against gliomas.^103^^,^^104^ Moreover, it acts synergistically with vMyx-IL15R***α***IL1 and adoptive GARC-1 tumor-specific CD8^+^ T-cells transfer and promotes CD8^+^ T cell infiltration by reducing Foxp3+Treg infiltration.^73^

#### Oclacitinib

Targets human JAK1/2 (Janus kinase) and is used to treat pruritus associated with allergic dermatitis and to control atopic dermatitis in dogs.^105^ By inhibiting JAK1, oclacitinib inhibits the IL-6 and IL-13 cytokines involved in inflammation and is predicted to inhibit type I IFN.^106^ Hence, MYXV replicates in cancer cells due to the damaged IFN responses; the effect of oclacitinib as an inhibitor of type I IFN should improve oncolysis.^70^ Ashton et al.^70^ demonstrated that the combination of MYXVΔ*SERP-2,* which has a deletion of a viral anti-apoptotic protein, prolonged viral replication in alveolar rhabdomyosarcoma allografts compared to viral monotherapy, but was not sufficient to improve outcomes, such as tumor growth rate or median survival time of the mice.

## MYXV targeted delivery into cancer cells

Despite the progress made in OVs engineering and enhancement, one of the main hurdles remains the delivery strategy, which is crucial for the success of the therapy in clinical application. OVs can be delivered by intratumoral injection or systemically by intravenous administration.^107^ Intratumoral delivery is an effective method, but there are some limitations. First, the tumor site is not always accessible.^108^ Second, the high interstitial pressure inside the tumor makes it difficult for the virus to penetrate.^109^ Third, cancer cells migrate to other organs, resulting in distant metastases, and the virus may not reach these sites easily. The solution to these limitations would be systemic delivery, as the virus would circulate throughout the body and infect distant tumors.^108^ At present, systemic delivery of free virus is not sufficiently efficient, mainly because of immune clearance, non-specific uptake by tissues such as the spleen or liver, poor ability of virus to cross the vascular compartment,^108^ blockade of the reticuloendothelial system (RES), and severe side effects.^109^

One way to deliver viruses is by using cells as carriers, by first infecting these carrier cells with the virus and then reinfusing the virus-adsorbed cells back into the cancer patient.^107^ The carrier cells approach prolongs drug circulation, enhances efficacy while minimizing immunogenicity and cytotoxicity, but it also serves as an ideal production factory. Different kinds of cells can be used: SCs, immune cells, and tumor cells.^109^ The mesenchymal stem cells (MSCs) and neural stem cells (NSCs) can be used to shield the virus from RES and provide protection against antiviral immune responses.^109^ The MSCs are pluripotent and are derived from bone marrow, pancreas, and adipose tissue and have the ability to differentiate into adipocytes and osteoblasts. They can also migrate towards an inflammatory microenvironment and engraft into the tumor stroma after systemic administration.^109^^,^^110^ In addition, they also exhibit low immunogenicity due to weak expression of MHC class I.^111^ NSCs are derived from or differentiated into neural tissue and have neuroprotective and neurotrophic functions. ADSCs have a natural chemotactic tropism for cancer tissues and can maintain their phenotype longer in culture and have a greater proliferative capacity compared to BM-MSCs. Another barrier to widespread use of MYXV is the inability of the virus to replicate in non-permissive cancer cell lines due to insufficient Akt phosphorylation. This could be overcome by incorporating additional viral proteins or by exploiting other oncogenic pathways that support replication. PDAC is extremely difficult to treat due to its poor immunogenicity and immunosuppressive tumor microenvironment, as well as its characteristic stromal fibroblast proliferation and ECM deposition, leading to a desmoplastic state.^94^

Jazowiecka-Rakus et al.^78^ used adipose-derived stem cells (ADSCs) as a Trojan horse to deliver a recombinant MYXV encoding the murine *LIGHT* gene (vMyx-*LIGHT*) in combination with gemcitabine to treat a mouse model of orthotopic PDAC lesion caused by Pan02 cells. Survival of mice treated with the «shielded» vMyx-*LIGHT* as monotherapy or in combination with gemcitabine was superior to that of mice treated with the unshielded vMyx-*LIGHT*, which may indicate that clearance of the unshielded virus is much faster. The shielded vMyx-*LIGHT* promoted the activation of the anti-tumor immune response, as evidenced by the increase in CD8^+^ up-regulation, and also increased the expression of innate response-mediating pro-inflammatory cytokines such as TNF-*α*, IFN-*γ*, IL-2, and IL-15 in pancreatic tissue compared to unshielded virus, suggesting effective penetration of the PDAC stroma. Interestingly, shielded vMyx-LIGHT monotherapy upregulated the expression of IL-10, which enables the expansion of tumor-resident CD8+ cells and may be involved in the maturation and differentiation of T cells in tumors exposed to oncolytics. These results confirm the advantage of the “Trojan horse” strategy used in this study.

In another study Jazowiecka-Rakus et al.^65^ used bone marrow-derived MSCs to deliver vMyx-IL15R**α**-tdTr to treat experimental murine lung melanoma. MSCs and melanoma cell lines are permissive to MYXV infection, and in the case of MSCs it does not induce significant changes in MHC class I expression, so it does not affect the immunogenicity of the cells. The authors concluded that MSC-shielded transfer of MYXV to B16-F10 melanoma cells occurs by cell-to-cell contact and not passively as with T lymphocytes. The difference between treatment with unshielded and shielded virus is that after the administration of the unshielded virus there was an increase in NK in the blood in the first 24 h, which did not happen with the shielded virus, suggesting that the shielded virus managed to evade the innate immune system, although after 48 h both viruses caused a 3-fold increase in NK in the lungs, which may be related to the transfer of virus between MSCs and melanoma cells. The protected virus persisted in the lungs for a long time, in contrast to the unprotected virus, which did not accumulate in the lungs. The adaptive anti-tumor immune response (presence of CD8^+^ and CD4^+^) in blood samples remained unchanged between the two treatments, but in the lungs CD8^+^ T cells increased 2.5-fold, which could be beneficial with the use of ICIs. IL-15, IFN-y, TNF-a, IL-1B, PD-1 and its ligand PD-L1 were upregulated after 21 days. The upregulation of CD8^+^ and cytokines suggests that there was an activation of the immune response, although this was counteracted by the upregulation of PD-1 and its ligand PD-L1, suggesting that melanoma may be sensitive to the use of ICIs. Human IFN-a had no significant effect on virus proliferation. MYXV replication in MSCs was much less robust, modest permissiveness, and largely unaffected viability, suggesting the suitability of MSCs for *in vivo* delivery of MYXV. They also found that the “naked” MYXV was cleared rapidly and did not accumulate in the lungs. The sclerotized septa and the absence of hyperemic areas in the lung sections of MSC-MYXV treatment are probably related to healed tumor foci. Treatment of tumors at other sites could be achieved by passage of MSCs into the arterial circulation after transit to the pulmonary microvasculature.

According to Villa et al.,^112^ different cell populations from BM or PBMC can be used as carriers of MYXV (e.g., T cells, neutrophils, NK cells, DCs, monocytes, and macrophages). It is interesting that the absence of B cells or NKs from BM and PBMC autographs did not impair the observed therapeutic effects, but the lack of lymphocytes significantly reduced the survival of mice. The researchers tried using autologous BM and PBMC to deliver MYXV to treat pre-seeded minimal residual disease (MRD) of MOPC315 BM cells. By using these carrier cells, there was an increase in survival rates and elimination of tumor burden, also mice seemed to have acquired immune protection against MM. This study suggests that myeloablative treatment can be supplemented *ex vivo* with oncolytic MYXV to eliminate MRD. In this study, they have observed that when the “naked” virus was injected, there was also an increase in the mice's survival rate. The authors suggest that the virus adhered to some lymphocytes that could facilitate the elimination of cancer cells.

MYXV can also bind to unstimulated human T lymphocytes, but efficient replication requires subsequent T cell activation with anti-CD3/CD28 beads.^113^ When cancer cells activate T cells, the MYXV can be delivered by T lymphocytes to tumor sites, where it can infect and kill malignant cells and potentially elicit an *in situ* vaccination effect against tumor antigens. This strategy may offer an alternative means of limiting antiviral immune responses, but it is constrained by relatively low viral replication and by restricted tumor accessibility for T lymphocytes.

Lilly et al.^64^ investigated the potential of *ex vivo* virotherapy with MYXV to eliminate residual MM disease in syngeneic immunocompetent BALB/c mice using the murine MM cell line MOPC315.BM, which has BM tropism and is similar to MM. MOPC315.BM cells are highly resistant to MYXV infection, making them difficult to treat *in vivo*. The authors treated donor murine C57BL/6 BM allografts with MYXV and then transplanted them into BALB/c mice bearing MOPC315.BM. The result was that almost all residual MOPC315.BM was eliminated, and it enhanced GVT against the pre-transplant disease. This showed that even “virus-resistant” cancers can be treated with oncolytic virotherapy. They also found that activated T cells and neutrophils from C57BL/6 allotransplanted with phorbol 12-myristate13-acetate (PMA) and ionomycin or anti-CD3/CD28 and treated with MYXV had a greater capacity to induce apoptosis in cancer cells. They concluded that neutrophils and T cells have the potential to be used as carrier cells after *ex vivo* treatment with MYXV and subsequent allotransplantation to eliminate cancer cells. The study also shows that onor leukocytes armed *ex vivo* with MYXV can deliver virus to tumor sites and promote elimination of residual disease *in vivo*, despite the target myeloma line being non-permissive to “naked” MYXV *in vitro*. The authors argue that the ability of MYXV-loaded BM grafts to nearly ablate residual myeloma (e.g., 17/19 spleens disease-free at <0.1% threshold) supports using this strategy whenever an allogeneic transplant is performed, to clear MRD.

Villa et al.^68^ used the C57BL/6 derived Vk∗MYC MM preclinical model, which more closely mimics the disease observed in human patients with MM. This model has low proliferation of monoclonal plasma cells (PCs) in their bone marrow and secondary lymphoid organs, resembling the features of monoclonal gammopathy of undetermined significance (MGUS)/MM. The authors injected bortezomib-resistant Vk12598 MM cells into mice and tested the potential of *ex vivo* autologous BM leukocyte cells pre-loaded with either unarmed vMyx-*M135KO* or armed vMyx-*hTNF v*iruses to treat MM. The results suggest that TNF-armed MYXV is a promising variant, but in key experiments, TNF did not improve survival over unarmed virus: BM+vMyx-*M135KO* 46.7% vs. BM+vMyx-hTNF 50.0% at day 90 (no statistically significant difference). In the Cy combination, BM/vMyx-*M135KO* gave 50.0% survival vs. 25.0% for BM/vMyx-*hTN*F and 25.0% for Cy alone, with no significant differences among cohorts II–IV; this pattern is at least compatible with TNF blunting benefit rather than enhancing it.^68^ Despite the encouraging re-challenge data, the sample sizes remain small and heterogeneous: 75% of mice from LCL161+α-PD-1+BM/vMyx-M135KO and 50% from LCL161+α-PD-1+BM/vMyx-hTNF survived 245 days after re-challenge, and 50% of LCL161+α-PD-1–only survivors also resisted re-challenge. The use of PBMC has also been tested in a mouse model of K7M2 lung metastatic osteosarcoma, which is highly resistant to wild-type MYXV infection. Recombinant MYXV vMyx-*hTNF*^98^ and vMyx-*mLIGHT*^79^ were used in combination with ICIs. As a result, mice lived longer, and tumor burden was reduced when these approaches were used compared to ICI monotherapy or virotherapy alone. Interestingly, although the free virus showed anti-tumor activity, it had to be administered four times in succession to achieve activity comparable to a single systemic dose of vMyx-hTNF/PBMC.^98^ These administration regimens should be further evaluated in terms of feasibility for clinical application.

## Comparison of MYXV with other oncolytic viruses

OVs use the natural life cycle of viruses to achieve selective cytotoxicity in cancer cells. They can be engineered to enhance their specificity, potency, and safety, and to induce robust anti-tumor immune responses.^114^ The selection of an optimal OV for therapeutic applications involves careful consideration of several criteria, including the size of the viral genome, transgene capacity, immunogenicity, pathogenicity, mechanism of cell entry, cell entry receptors, and the ability to penetrate the BBB. We summarize selected criteria for optimal oncolytic usage (Table 2) and justify their significance.

**Table 2:** Comparative analysis of myxoma virus oncolytic properties with other viruses

Virus	Genome size	Cell entry mechanism	Cell entry receptors	Transgene capacity	Immunogenicity	Ability to penetrate BBB	Pathogenicity to humans	Current highest clinical trial phase	Manufacturing scalability	Reference
MYXV	160–161.8 kb	endocytosis Membrane fusion Receptors? Cholesterol?	Uknown	high	high	limited	non-pathogenic	preclinical	moderate	^3^^,^^46^^,^^115^
HSV-1 (T-VEC, Talimogene laherparepvec, Imlygic®) HSV-1 (Teserpaturev/G47Δ)	150-152 kb	endocytosis; penetration	HVEM, nectin 1; nectin 2	high	low	limited	cold sore and/or fever blister; latent infection in the CNS possible	approved	high	^1^^,^^116^
Adenovirus (H101, Oncorine®)	34-36 kb	endocytosis	hCAR; VCAM1; CD46	moderate	low	limited	respiratory and GI tract infections; pharyngitis; pneumonia; meningitis; follicular conjunctivitis	approved	high	^117^^,^^118^^,^^119^
Enterovirus (Coxsakie virus A21, Cavatak)	7–8.5 kb	micropinocytosis vía epithelial tight junctions, receptor-mediated endocytosis	CAR; DAF, CD155	low	low-moderate	moderate-high	common cold; HFM disease; pleurodynia; myocarditis, Poliomyelitis	phase II	moderate	^120^^,^^121^^,^^122^^,^^123^
NDV	15-16 kb	membrane fusion	neuraminidase receptor; sialoglyco conjugates	low	low	very limited	transitory conjunctivitis	phase I	moderate	^124^^,^^125^
Reovirus (Reolysin)	18-24 kb	receptor-mediated endocytosis	JAM-A	low	moderate	moderate	generally non-pathogenic but could cause mild respiratory and gastrointestinal diseases in children and could cause severe diseases in immunocompromised individuals	phase III	high	^126^^,^^127^^,^^128^^,^^129^
Measles virus	15-16 kb	membrane fusion	CD46; SLAM	low	low	high	Measles, pneumonia	phase II	moderate	^130^^,^^131^^,^^132^^,^^133^
Poxvirus (Vaccinia virus)	130–360 kb	membrane penetration and fusion	GAGs; EFC	high	high	very limited	Fever, myalgia, lymphadenopathy; eczema; generalized infection.	phase II	high	^24^^,^^134^
VSV	11–11.2 kb	clathrin-dependent endocytosis	low-density Lipoprotein receptor	moderate	moderate	limited- high	Generally non-pathogenic in humans but cause mild flu-like symptoms like headache, fever, myalgia, weakness. Rarely oral blisters.	phase II	high	^135^^,^^136^^,^^137^

### Viral genome

The genome size is crucial in selecting and optimizing OVs for therapeutic applications. Large-genome viruses such as poxviruses (MYXV, vaccinia virus) and herpesviruses (HSV) offer significant advantages in genetic engineering and therapeutic gene delivery.^21^ Viruses with large genomes can incorporate multiple therapeutic genes, providing flexibility in genetic engineering. Viruses with moderate-sized genomes offer a balance between carrying capacity and ease of engineering. Viruses with small genomes (<5 kb) have limited capacity for additional genes.^114^^,^^138^ DNA viruses are typically easier to genetically engineer, although RNA viruses might be more challenging to engineer, they replicate more efficiently, thus resulting in greater local amplification.^21^^,^^139^ Examples of viruses with large genomes (>100 kbp) include MYXV, vaccinia virus, and HSV, while those with moderate genomes (between 100 kb and 10 kb) include adenovirus, reovirus, NDV, vesicular stomatitis virus (VSV), measles virus, and those with relatively small genomes include enteroviruses and adeno-associated virus (<10 kb).^21^^,^^140^

### Cell entry mechanism

The cell entry mechanism is critical in determining the oncolytic efficacy of viruses. Understanding the mechanisms of cell entry is crucial for the optimization of OVs. The cell entry mechanism determines the ability of the virus to infect and lyse cancer cells effectively.^133^^,^^141^^,^^142^ Cell entry receptors are specific molecules on the surface of cells that viruses bind to gain entry into the host cell. These receptors are crucial for determining the tropism of the virus. Understanding which receptors a virus uses to enter cells helps in selecting and designing viruses that can specifically target and kill cancer cells.^143^^,^^144^^,^^145^

### Immunogenicity

Immunogenicity refers to a virus’s ability to stimulate an immune response, involving both the innate and adaptive immune systems. While viruses are generally potent activators of these immune responses, the strength of this effect can vary. Immunogenicity is a critical factor that shapes therapeutic strategies, including the choice of administration route and dosing regimen. Viruses that elicit a strong immune response may promote robust antitumor immunity but can also be cleared rapidly by the immune system, limiting the number of doses that can be administered systemically. Therefore, viruses with higher immunogenicity are often better suited for intratumoral delivery, where localized immune activation is desirable. In contrast, viruses with lower immunogenicity may persist longer in the body and potentially are more effective for intravenous delivery.^141^^,^^142^^,^^146^

In fact, MYXV can efficiently suppress both innate and adaptive antiviral immune responses through its arsenal of immunomodulatory proteins in its natural hosts. Nevertheless, MYXV is also known for triggering a strong immune response at the late time points upon infection.

### Ability to penetrate the blood brain barrier (BBB)

The ability of OVs to penetrate the BBB is an important factor, especially when targeting primary brain tumors or brain metastases. However, penetration might be undesirable if patients have extracranial tumors and/or if there might be central nervous system toxicity associated with viral infection. The ability of a virus to cross this barrier can significantly impact its therapeutic potential and safety profile. Viruses with high ability efficiently cross the BBB and are suitable for treating brain tumors, while those with moderate ability can penetrate the BBB under certain conditions, and those with limited ability have poor penetration.^142^^,^^147^

### Pathogenicity

The pathogenicity of the virus is the ability of the virus to cause disease in humans. There is no scientific evidence demonstrating MYXV's ability to cause disease in humans. High pathogenicity of some viruses (e.g., HSV, AdV, and ZIKA virus) could limit their clinical use; hence, less pathogenic viruses are preferred for clinical use, especially if attenuated. In this case, deletion of virulence genes can enhance safety.^146^^,^^148^

In summary, based on the criteria outlined above, MYXV offers significant advantages in oncolytic virotherapy due to its large genome size (160–161.8 kbp), allowing it to carry multiple transgenes without compromising its ability to replicate.^3^^,^^149^^,^^150^ It shows high transgene capacity, making it a flexible platform for genetic engineering. Additionally, its non-pathogenicity in humans adds to its safety profile, as it does not cause disease in humans or other non-lagomorphs.^3^ Compared to other viruses such as adenovirus and HSV, which may have limitations in transgene capacity or induce lower immune responses, MYXV stands out for its broad applicability across various cancer types. However, its restricted ability to penetrate the BBB limits its application for brain tumors treatment. Future strategies to enhance MYXV’s efficacy may involve nanoparticle-mediated delivery systems, shielding MYXV with BM or PBMCs, or combinatorial therapies with approved anti-cancer drugs that.^151^

## Discussion

**Figure 3:**
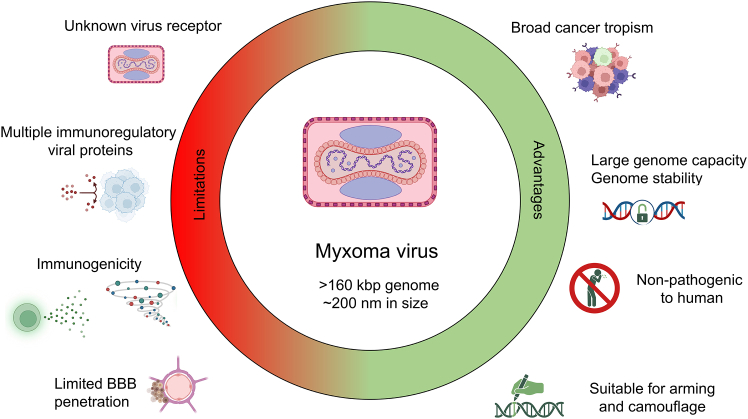
Schematic diagram depicting major characteristics of oncolytic MYXV Limitations: (1) relatively large size of MYXV may limit its penetration in tissues; (2) despite broad tropism to various cancer types, not all malignant cells are equally susceptible to MYXV (i.e., pancreatic cancer cell lines (PAC-1); and (3) Unknown virus receptors possess potential risk for off-target effect of immunotherapy. Advantages: (1) MYXV, like many other double-stranded DNA viruses, has high genome stability; (2) MYXV genome is 160 kb long, has many intergenic non-coding regions allowing seamless cloning of multiple transgenes; and (3) MYXV can be armed with additional molecules enhancing virus specificity and bioavailability. Moreover, MYXV can be delivered inside autologous cells to protect the virus for host immune response.

OVs have been extensively studied for decades, and hundreds of clinical trials have been conducted with some therapeutic success. Recent advances in molecular virology and the need for novel viral vectors have stimulated the search for animal OVs with therapeutic potential. Accumulating scientific evidence and recent progress in cancer immunotherapy suggest that the MYXV is a promising OV. We summarize some important MYXV features in Figure 3 depicting pros and cons as a potential viral vector for cancer immunotherapy (Figure 3).

The tropism of MYXV is strictly limited to the rabbit species and has never been associated with human pathology, making it safe for humans. Interestingly, MYXV has the ability to replicate in human cancer cells due to inactive tumor suppressor pathways.

Although the specific cellular receptors for MYXV attachment are currently unknown, several determinants of binding on the cell surface (heparin sulfate, chondroitin sulfate, GAGs) have been identified. For a deep understanding of the mechanisms of MYXV binding and penetration into the cell, as well as to avoid off-target effects, it is necessary to study these receptors in detail using modern biophysical methods. For example, confocal microscopy can detect the colocalization of a potential receptor with viral particles, as shown in the work of Griffiths and colleagues about RSV, the insulin-like growth factor-1 receptor (IGF1R) and its coreceptor, nucleolin (NCL).^152^ Using fluorescence microscopy and lipophilic dyes such as DiD, DiL, DiO, and DiR, it is possible to label membranes and viral envelopes and observe the process of cell infection in real time.^153^ In addition, the method of imaging flow cytometry (IFC), combining the principles of fluorescence microscopy and flow cytometry, allows us to characterize the interaction between the host cell and the virus and collect statistically reliable data.^152^^,^^154^ Using IFC, for example, the effect of syndecans, a family of transmembrane proteoglycans, on the penetration of SARS-CoV-2 into cells was evaluated.^155^ Cryogenic electron microscopy (cryo-EM) is a powerful method for determining the structure and conformation of proteins in their native state, which determines the application of this method in structural virology studies.^156^ A very recent study using cryo-EM and AlphaFold modeling determined the structure of the vaccinia virus entry fusion complex, which includes 15 proteins.^157^ Another method for conducting quantitative analysis of molecular interactions in real time is surface plasmon resonance (SPR). Using SPR, it was demonstrated how mpox virus protein A29 (homolog of vaccinia virus protein A27) binds to GAG.^158^ The microscale thermophoresis (MT) method, based on the movement of molecules in a temperature gradient, allowed us to evaluate the binding of the receptor binding domain (RBD) of the SARS-CoV-2 spike protein to the ACE2 viral receptor.^159^

The MYXV genome can accommodate multiple payloads (e.g., miRNA, shRNA, genes encoding cancer-specific antigens, cytokines, ICIs, and so forth) without compromising viral replication. The MYXV DNA genome is stable and has a low mutation rate, making it highly adaptable for genetic engineering and cancer therapy. MYXV can also be used as a vehicle for gene editing cancer therapy to deliver gene editing tools. Recombinant vMyx-SpCas9-2A-Csy4 has demonstrated enhanced anti-cancer activity (reducing tumor growth and increasing OS) in a human embryonal rhabdomyosarcoma xenograft model.^148^ The study suggests that the use of MYXV as a promising therapeutic delivery vehicle can be expanded.

We have found 61 studies testing MYXV in different mouse models. More than half of them (54%) used it as a monotherapy (Table S1). Combinatorial approaches with chemotherapeutics, ICIs, or other drugs are less common (16%,14%, and 13%, respectively). Recent studies also demonstrate some promising combinations of MYXV and T cell therapy. However, the number of studies is still limited. MYXV is preferentially administered by direct intratumoral injections (60%), followed by retroorbital (16%) and systemic (9%) inoculation (see Table S1). The intratumoral route of administration is likely to be chosen because of the type of cancer studied in the mouse model, accessibility, and the ability to control the virus dose.

Despite the fact that the systemic route is more preferable in clinical settings, animal studies with MYXV suggest its limited efficacy. Oncolytic MYXV has several other practical limitations. Due to its large size (>200 nm), MYXV has a limited ability to penetrate the BBB. This is a challenge for the treatment of brain tumors. This obstacle could be overcome by improving delivery, e.g., by using carrier cells, SCs, and immune cells, which would minimize immunogenicity and cytotoxicity and act as production factories. Another barrier to widespread use of MYXV is the inability of the virus to replicate in non-permissive cancer cell lines due to insufficient Akt phosphorylation. This could be overcome by incorporating additional viral proteins or by exploiting other oncogenic pathways that support replication. Promising preclinical mouse studies suggest that MYXV is well tolerated, non-pathogenic, and less immunogenic than other OVs (Table 2). Nevertheless, rodent and rabbit immune systems are different from those of humans, particularly in the context of anti-viral immune response. MYXV has an extremely narrow host range and causes severe immunosuppression in rabbits that is not expected in primates or humans. Thorough interspecies studies will be crucial to further support the translation of MYXV to clinical trials.

MYXV efficiently suppresses both innate and adaptive antiviral immune responses through its arsenal of immunomodulatory proteins in its natural hosts. One of the mechanisms by which MYXV dampens the immune response is by reducing the expression of MHC-I on the surface of infected cells.^160^^,^^161^ By downregulating MHC-I and thereby inhibiting the presentation of viral antigens, MYXV prevents virus-specific cytotoxic T lymphocytes from recognizing infected cells. This allows MYXV to establish infection during the early stages following cell entry. In the context of infected cancer cells, MYXV’s immunomodulatory proteins provide the virus with an advantage by evading early antiviral immune responses.

Nevertheless, MYXV cannot fully suppress antiviral responses in non-permissive hosts (e.g., humans and mice) and eventually triggers strong proinflammatory activation. MYXV partially induces IFN responses via RIG-I and fails to evade pre-induced antiviral states. In addition, host effectors such as SAMD9 can induce proinflammatory responses through the cGAS-STING pathway. Although MYXV elicits an antiviral immune response in the tumor microenvironment at later stages, this effect may actually enhance combinatorial immunotherapy by providing additional activation signals to cytotoxic T cells and potentially reshaping the TIME.

**Figure 4:**
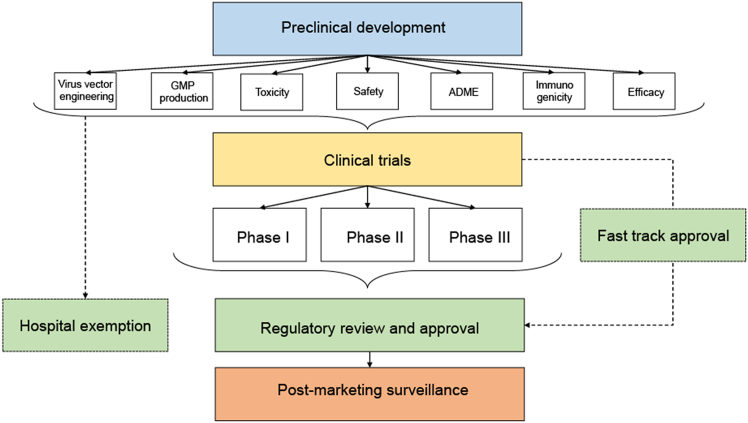
Schematic representation of the logical framework for the MYXV transition for preclinical development to clinical trials Preclinical development phases include virus vector engineering and establishing a controlled GMP manufacturing process. A comprehensive non-clinical safety and toxicity study (i.e., general toxicity, carcinogenicity, genotoxicity, and reproductive and developmental toxicity), pharmacokinetic studies (absorption, distribution, metabolism, and excretion (ADME)), immunogenicity, and efficacy using an *in vivo* model are critical before human studies can begin.^164^ Clinical trials run in three main phases (I, II, III), aiming to assess safety, dosing, biological activity, and clinical benefits in humans. Detailed data from preclinical and clinical studies are submitted according to either ICH, FDA, or EMA guidelines for regulatory review and approval^165^

In order to ensure safety, efficacy, and consistency of MYXV batch production, it is essential to implement rigorous and multifaceted quality control measures. The European Medicines Agency (EMA) asserts that oncolytics are classified as Advanced Therapy Medicinal Products (ATMPs) or biological gene therapy products according to the FDA guideline. A comprehensive list of quality control measures is recommended, including identity testing, purity and sterility, potency, safety risk management, manufacturing consistency, and regulatory compliance (Figure 4). Readers may also find specific information and requirements in EMA and FDA guidelines on quality, non-clinical, and clinical requirements for investigational advanced therapy medicinal products in clinical trials.^162^^,^^163^

Evidence from veterinary vaccine manufacturers using MYXV as a vaccine against myxomatosis indicates that the production of MYXV can be readily scaled up, and MYXV oncolytic can be produced according to the GMP in order to meet the requisite dosage and quality standards for human clinical trials. As demonstrated by our group^166^ and others^167^ attenuated and cell culture-adapted MYXV vaccine strains have the capacity to replicate in a rabbit kidney cell line (i.e., RK-13), achieving high titers (>1.0 + 09E TCID50/mL), without compromising the integrity of the virus genome, its proliferative and oncolytic potential. Moreover, recombinant proapoptotic MYXV virus lacking the M011L gene can reach even higher titers in RK-13 cells (Isaeva et al., unpublished data) and therefore be a strong candidate for clinical translation.^69^ Post-marketing surveillance is an important step for the ATMP to assess long-term efficacy, safety, and adverse effects. According to the EMA the ATMPs can be approved for clinical use without conventional clinical trials or authorization if they meet “hospital exemption” criteria. The products still must meet strict quality standards and be manufactured in accordance with GMP practices. In some cases, the ATMPs can be granted a fast track or breakthrough approval if substantial improvements are shown in early clinical trial phase over existing options for serious medical conditions (e.g., genetic disorders). Fast-track designation using preliminary clinical data may significantly accelerate drug development and save the lives of sick individuals. The MYXV oncolytic transition to clinical trials is significantly delayed by the absence of human safety information and immune characterization of the virus in other species.^2^^,^^168^ Despite the fact that MYXV is a well-known leporipoxvirus infecting only lagomorphs, its oncolytic potential has only been discovered relatively recently.^2^ The mounting body of scientific evidence, coupled with the increasing number of translation research using MYXV-based virotherapy, will ultimately facilitate the accumulation of substantial knowledge, paving the way for more extensive preclinical studies and eventual transition to clinical trials. Despite the fact that there are no clinical trials using oncolytic MYXV, we made a cautious attempt to propose some primary endpoints for human clinical studies with MYXV. In accordance with the aforementioned EMA/FDA guidelines, the primary endpoints should directly demonstrate a clinically meaningful benefit to the patient, such as OS. Secondary endpoints, including progression-free survival (PFS), disease-free survival (DFS), and health-related quality of life (HRQoL), support the primary goal or measure additional benefits. It is evident that tertiary or exploratory endpoints have the capacity to provide further insight into the agent’s mechanism or to investigate alternative outcomes.^169^^,^^170^

Potential primary endpoints for Phase I clinical trials for MYXV must be focused on establishing a safety profile for MYXV. Therefore, dose-limiting toxicities (DLTs) are the desirable primary endpoint, assessing the drug’s safety. In later clinical trials, the durable response rate (DRR) is a primary endpoint that measures the percentage of patients with a sustained benefit from the treatment that could be used.^171^ Additional suggestions have been proposed elsewhere by leading researchers in the field.^2^ In addition, we have previously reviewed clinical studies using OVs, and we refer readers seeking additional information to this review.^6^ The searchable table provides a comprehensive overview of available data from various oncolytic clinical trials, allowing readers to filter the data according to specific criteria, including cancer type, dosage, adverse effects, and combinations with other anti-cancer drugs.

It is worth noting that a number of studies have indicated the presence of certain concerning limitations in the efficacy of oncolytic therapy. Preclinical studies of VSV-IFNβ in HCC mouse models demonstrate a robust anti-viral CD8^+^ T cell response but a weaker anti-tumor T cell response, even when supplemented with anti-PD-L1 therapy. The data suggests a potential interference between these two responses, which may consequently exert a negative regulatory effect on the formation of anti-cancer memory T cells. Consequently, the authors hypothesize that engineering the virus to express TAAs may enhance anti-tumor T cell responses, thus optimizing combinatorial therapy, including ICI.^172^

It is also noteworthy that similar conclusions have been announced in preclinical studies employing the approved oncolytic T-VEC and melanoma mouse model. The authors confirmed the presence of viral antigen-specific T cells in virus-injected tumors. These cells were in a phenotypic state that would enable them to mount an effective response against virally infected cells. Of particular significance is the demonstration of evidence derived from human peripheral blood samples that were either treated or not treated with T-VEC. The absence of an anti-cancer antigen response occurs despite enhanced cancer antigen presentation following OV delivery.^173^ The data suggested that the effective delivery of viruses for immunotherapy needs meticulous consideration. One potential solution to the issue of unwanted priming of immune cells with viral antigens could be the packaging of OVs in nanoparticles (e.g., biological nanoparticles and microvesicles) or the loading of transporter cells *in vitro*.^109^^,^^174^ As an alternative option, the utilization of less immunogenic viral capsids (e.g., recombinant AAV, SV40, and so forth) may also assist in the mitigation of the risk of a strong and interfering antiviral immune response.^175^

Overall, MYXV offers significant advantages for next-generation oncolytic virotherapy, particularly due to its genetic flexibility and safety profile. However, overcoming its current limitations by using it in combination with other therapies such as chemotherapy, immunotherapy (ICIs and ATCs) would significantly expand its therapeutic potential and efficacy in cancer treatment.

The completion of preclinical milestones and the development of safe delivery methods will facilitate a seamless transition of MYXV to clinical trials.

## Acknowledgments

The Molecular virology laboratory is supported by the Academic leadership program Priority 2030 by the Federal State Autonomous Educational Institution of Higher Education I.M. Sechenov, First Moscow State Medical University of the Ministry of Health of the Russian Federation (Sechenov University). We thank colleagues at the Federal Research Center for Virology and Microbiology for providing valuable information about MYXV history. Figures were created using BioRender.com (2025) templates with our modifications.

## Author contributions

T.Y.A.D.: writing the first draft, I.A.S.: writing the first draft and drawing the figures, I.E.E.: writing and reviewing the manuscript, Z.S.P.: writing and reviewing the manuscript, Y.S.G.: writing and reviewing the manuscript, M.A.S.: conceptualization, drawing the figures and editing the manuscript.

## Declaration of interests

The authors declare no conflict of interests.
