# Supplementary material for: Oncolytic myxoma virus and engineering strategies for advanced cancer immunotherapy
Source: iScience. 2026 Apr 20;29(6):115812. doi: 10.1016/j.isci.2026.115812 (PMC13194175; doi:10.1016/j.isci.2026.115812)
Supplement: Document S1. Table S1 [file mmc1.pdf]

iScience, Volume *29*

Supplemental information

Oncolytic myxoma virus and engineering strategies for advanced cancer immunotherapy

A.D. Trujillo Yeriomenko, A.S. Isaeva, E.E. Idota, S.P. Zhivoderov, S.G. Yurkov, and A.S. Malogolovkin

Adminitration route	MYXV Modification	Shielded/Naked MYXV	Cancer type	Tumor Establishment	Mouse model	Type of therapy (Combo/Mono)	Results	Reference
Intranasal	*vMyx-M135KO-gfp*	Naked	Small cell lung cancer SCLC	Intratracheal injection	C57BL/6 (p53lox/loxP p130lox2722/lox2722 RbloxP/loxP)	Combo (Cisplatin)	In vivo mouse studies showed that MYXV, including *vMyx-M135KO-GFP* , in combination with cisplatin treatment resulted in efficient infection and late viral replication in human SCLC cell lines, independent of cell morphology phenotype. The primary human SCLC specimen infected with vMyx-M135KO-GFP demonstrated viral replication and extensive tumor necrosis after direct intratumoral delivery, indicating the effectiveness of the virus in targeting SCLC tumors	67
Intratumoral	*vMyx-GFP-tdTr*	Naked	Small cell lung cancer SCLC	Subcutaneously	NSG	Mono	Virus replication and extensive tumor necrosis	
Intratumoral	*vMyx-FLuc*	Naked	Small cell lung cancer SCLC	Subcutaneously	C57BL/6	Mono	Virus replication, extensive tumor necrosis and CD45+ immune cell infiltration	
Intraperitoneal	*vMyx-GFP*	Naked	Ovarian cancer	Intraperitoneal cavity	C57BL/6	Combo (Cisplatin)	The combinatorial treatment using first MYXV and after cisplatin (100% survival) was more effective than the other way around (30% survival). MYXV can suppress STAT3 and AKT signaling.	63
Intraperitoneal	*vMyx-M062RKO*	Naked	Ovarian cancer	Intraperitoneal cavity	C57BL/6	Cisplatin	The combinatorial treatment using first vMyx*M062RKO* and after cisplatin (90% survival) was more effective than the other way around (60% survival). The suppression of STAT3 and AKT signaling can be enhanced using this construct. The vMyx*M062RKO* reduces phosphorylated CREB.	
Intratumoral	*vMyx-WT*	Naked	Glioblastoma	Intracranial injection	C57BL/6	Temozolomide	The vMyx-WT failed to induce apoptosis as M011L protein prevented Bax activation and cytochrome c release.	69
Intratumoral	*vMyx-M11KO*	Naked	Glioblastoma	Intracranial injection	C57BL/6	Temozolomide	The vMyx-*M011L* -KO induces apoptosis in BTICs, increase sensitivity to the virus. Prolonged survival in immunocompetent but not immunodeficient mouse models.	
Intratumoral	*vMyx-GFP*	Naked	Gallbladder cancer	Subcutaneously	CD-1 Naked	Rapamycin	MYXV + Rap treatment increased the oncolytic ability of MYXV against GBC cell lines in vitro. The authors found higher levels of collagen IV in GBC tumors than in glioma tumors. Diffusion analysis demonstrated that collagen IV could physically hinder MYXV intratumoral distribution.	99
Intratumoral	*vMyx-GFP*	Naked	Gallbladder cancer	Subcutaneously	CD-1 Naked	Hyaluronan	HA–CD44 interplay was found to activate the Akt signaling pathway, which increases oncolytic rates. HA was also found to enhance the MMP-9 secretion, which contributes to collagen IV degradation.	
Intratumoral	*vMyx-tdTr*	Naked	Melanoma	Intracranial injection	C57BL/6 and C57BL/6 RAG1-/-	Monotherapy	Robust expression of tdTomato red protein in the tumors, but not in surrounding normal brain	97
Intratumoral	*vMyx-tdTr*	Naked	Melanoma	Intracranial injection	C57BL/6 and C57BL/6 RAG1-/-	Activated T cells (2C T cells)+Abs (neutralizing antibodies of IFN B	Mice that received the triple combination treatment lived three times longer than untreated mice	
Intratumoral	*vMyx-tdTr*	Naked	Melanoma	Intracranial injection	C57BL/6 and C57BL/6 RAG1-/-	Rapamycin	Mice that received both MYXV-Red and rapamycin lived significantly longer	
Intratumoral	*vMyx-tdTr*	Naked	Melanoma	Intracranial injection	C57BL/6 and C57BL/6 RAG1-/-	Rapamycin + 2C T cells	Mice receiving the triple combination treatment lived significantly longer than mice receiving only T cells, and the combination of virus and rapamycin was required for the survival benefit	
Intratumoral	*vMyx-Tred*	Naked	Melanoma	Subcutaneously	C57BL/6	Monotherapy	Prolonged survival of mice treated with vMyx-tred	71
Intratumoral	*vMyx-IL-15*	Naked	Melanoma	Subcutaneously	C57BL/6		Prolonged survival of mice treated with vMyx-IL15	
Intratumoral	*vMyx-IL15Rα-tdTr*	Naked	Melanoma	Subcutaneously	C57BL/6 and C57BL/6 RAG1-/-	Monotherapy	Attenuated tumor growth and higher survival benefit, increased infiltration of NK cells and CD3+ cells, by adding IL15Rα fusion protein improves IL15 effects	72

Systemic	*vMyx-M135KO-GFP*	Bone marrow	Multiple myeloma	Intravenously	BALB/c	Monotherapy	Ex vivo virotherapy with MYXV arms cells within a donor allotransplant resulting in a dramatic positive enhancement of GVT in a model where the pre-seeded residual target tumor cell is highly resistant to direct infection by free virus Prolonged survival of mice	64
Systemic	*vMyx-GFP*	Naked	Multiple myeloma	Intravenously	BALB/c	Monotherapy	Acute elimination of the majority of residual MM cells from Bone Marrow, apparent eradication of clinical disease, increased survival in mice with established residual MM	74
Intratumoral	*vMyx-SpCas9-2A-Csy4*	Naked	Embryonal rhabdomyosarcoma	Subcutaneously	NSG	Monotherapy	The vector was capable of replicating within ERMS cells, efficiently edits endogenous human genes using single SpCas9-Csy4 transcript with cleavable gRNAs	148
Intratumoral	*vMyxASS1-GFP*	Naked	Melanoma	Subcutaneously	C57B1/6			
Intratumoral	*vMyxASS1FS-GFP*	Naked	Melanoma	Subcutaneously	C57B1/6	Monotherapy	The paper demonstrates that intratumoral defects in arginine metabolism can act as a barrier to virally induced immunotherapy, specifically oncolytic virotherapy (OV) using myxoma virus (MYXV). The exogenous expression of ASS1 from recombinant MYXV constructs partially rescues the defects in MYXV replication and therapeutic responses in ASS1-deficient tumors, suggesting that virally reconstituting arginine biosynthesis can improve the efficacy of OV in arginine-auxotrophic tumors	
Intratumoral	*vMyxPD1/IL--12/ASS1*	Naked	Melanoma	Subcutaneously	C57B1/6			74
Intratumoral	*vMyx-WT*	Naked	Melanoma	Subcutaneously	C57/B6	TIM3	Combination therapy completely eradicated disease in ~30% of mice. Additionally, even in mice who did not display a complete remission, the combination of MYXV and αTIM3 antibody still improved overall survival and reduced tumor burden	
Intratumoral	*vMyxPD1/IL12*	Naked	Triple negative breast cancer	Implantation orthotopically into the fourth left mammary fat pad	BALBc	Monotherapy	vMyxPD1/IL12-treated mice displayed significantly delayed tumor growth, decreases lung metastases and improves survival of mice bearing metastasic 4T1 tumors	75
Intratumoral	*VMyx-Fluc*	Naked	colorectal adenocarcinoma	Subcutaneously	NSG	Monotherapy	Reduced tumor burden	
Intratumoral	*VMyx-Fluc*	Naked	epithelioid carcinoma	Subcutaneously	NSG			
Intratumoral	*VMyx-Fluc*	Naked	adenocarcinoma colorectal	Subcutaneously	NSG	Selinexor	Reduced tumor burden compared to Monotherapy in tumors (Colo205 and HT29), Selinexor enhanced MYXV gene expression and replication in vivo. In xenograft PANC-1 model was observed reduction of tumor burden compared to the Monotherapy, the survival rate was significantly higher than the treated only with selinexor alone	102
Intratumoral	*VMyx-Fluc*	Naked	epithelioid carcinoma	Subcutaneously	NSG		Reduced tumor burden compared to Monotherapy in tumors (Colo205 and HT29), Selinexor enhanced MYXV gene expression and replication in vivo. In xenograft PANC-1 model was observed reduction of tumor burden compared to the Monotherapy, the survival rate was significantly higher than the treated only with selinexor alone	
Intratumoral	*vMyx-IFNy*	Naked	Melanoma	Subcutaneously	C57B16/J	Monotherapy	No data	
Intratumoral	*vMyx-IFNy*	Naked	Melanoma	Subcutaneously	C57B16/J	α-PDL1	Reduction of tumour growth, but didn't improve further the tumor growth inhibition compaired with vMyx-mCherry with aPD-L1, increase in CD3+ and CD8+ T cells, but didn't show increase in TIL compared to the aPD-L1 single treatment group	
Intratumoral	*vMyx-CD47*	Naked	Melanoma	Subcutaneously	C57B16/J	Monotherapy	More CD47+ cells than the present after the treatment with vMyx-IFNy, and increased IFNy+CD4+T cell population in the tumor	
Intratumoral	*vMyx-CD47*	Naked	Melanoma	Subcutaneously	C57B16/J	α-PDL1	reduction on tumour growth, had the strongest tumor growth inhibitory activity, increase in CD3+ and CD8+ T cells, but i didn't show increase in TIL compared to the aPD-L1 single treatment group	76
Intratumoral	*vMyx-Cd47/IFNy*	Naked	Melanoma	Subcutaneously	C57B16/J	Monotherapy	Anticancer efficacy with average tumor growth inhibitions of 84.1% compared to control group and 69.6%, 73.6% and 68.4% compared to vMyx-mCherry, vMyx-CD47 and vMyx-IFNy. It was seen an increase of CD3+ T cells and CD8+T cells, also an increase of IFNy+ CD8+ and Granzyme b+CD8+ T cell	
Intratumoral	*vMyx-Cd47/IFNy*	Naked	Melanoma	Subcutaneously	C57B16/J	α-PDL1	reduction on tumour growth, this combined treatment showed significant tumor growth inhibition compaired with vMyx-mcherry, increase in CD3+ and CD8+ T cells. the percentage of activated T cells, IFNy+CD4+T cells increased in tumor infiltrated CD3+Tcell population.	
Intratumoral	*vMyx-PD1-IL12*	Naked	Melanoma	Subcutaneously	C57B16, RAG-/- and NSG			
Intratumoral	*vMyx-PD1-IL12*	Naked	lewis lung carcinoma	Subcutaneously	C57B16, RAG-/- and NSG	Monotherapy	Regression of dissease. In LLC and BR5 models, there was a complete phenotypic elimnation of all tumors and long term survival. In B16F10 and MC38 models there was an improvement in overall survival, but non curative. The elimination of T cells restricts the efficacy of vMyxPD1/IL12 eliminating all long therm phenotypic regressions in LLC and BR5 models. The curative responses are dependent on functional T cell immunity	
Intratumoral	*vMyx-PD1-IL12*	Naked	colon adenocarcinoma	Subcutaneously	C57B16, RAG-/- and NSG			74

							and BR5 models. The curative responses are dependent on functional T cell immunity.	74
Intratumoral	*vMyx-PD1-IL12*	Naked	ovarian cancer	Subcutaneously	C57B16, RAG-/- and NSG			
Intratumoral	*vMyx-PD1-IL12*	Naked	Melanoma	Subcutaneously	TNG-/-	TNF-blocking antibody	This treatment allowed to occur curative responses in B16F10 and MC38 models, the virus induced more regulatory T cells, increase of viable CD8+ T cells	
Intratumoral	*vMyx-PD1-IL12*	Naked	colon adenocarcinoma	Subcutaneously	TNG-/-	TNF-blocking antibody		
Intraperitoneal	*vMyx-mLIGHT-FLuc/tdTr*	Naked	Pancreatic ductal adenocarcinoma	Abdominal surgery	C57B1/6NCrl	Monotherapy	Rich cell infiltration in pancreatic specimens, quicker clearence of the oncolytic therapeutic, survival of 14%. There was a modulation of the immune microenvironment in the tissues bur more with ADSC shielded virus	
Intraperitoneal	*vMyx-mLIGHT-FLuc/tdTr*	Naked	Pancreatic ductal adenocarcinoma	Abdominal surgery	C57B1/6NCrl	Gemcitabine	Rich cell infiltration in pancreatic specimens, quicker clearence of the oncolytic therapeutic, gemcitabine showed a detrimental effect.	
Intraperitoneal	*vMyx-mLIGHT-FLuc/tdTr*	Adipose derived stem cells	Pancreatic ductal adenocarcinoma	Abdominal surgery	C57B1/6NCrl	Monotherapy	Strong lymphocytic infiltrates and fibrosis in pancreatic specimens, survival was longer than the other treatments (50%). The ADSCs packaging imrpove the survival of virus cargo during transi and its delivery to the PDAC lesions before immune clearance. There was a modulation of the immune microenvironment in the tissues bur more with ADSC shielded virus. There was an increase in pro inflammatory cytokines (NF-a, IFNy, IL-2 and IL 15)	78
Intraperitoneal	*vMyx-mLIGHT-FLuc/tdTr*	Adipose derived stem cells	Pancreatic ductal adenocarcinoma	Abdominal surgery	C57B1/6NCrl	Gemcitabine	Strong lymphocytic infiltrates and fibrosis in pancreatic specimens, survival was longer. The ADSCs packaging imrpove the survival of virus cargo during transi and its delivery to the PDAC lesions before immune clearance	
Retro-orbital/systemic	*vMyx-M135KO-GFP*	Naked	Multiple myeloma	Intravenously	C57BL/6	Monotherapy	The disease was deleyed, survival rates increased, the BM ex vivo loaded virus had superior therapeutic effects compared to the free virus	
Retro-orbital/systemic	*vMyx-M135KO-GFP*	Bone marrow leukocytes	Multiple myeloma	Intravenously	C57BL/6	Monotherapy		
Retro-orbital/systemic	*vMyx-M135KO-GFP*	Bone marrow leukocytes	Multiple myeloma	Intravenously	C57BL/6	Cyclophosphamide	There was no significant difference in the deleay of the disease between the group treated only with cyclophosphamide	
Retro-orbital/systemic	*vMyx-M135KO-GFP*	Bone marrow leukocytes	Multiple myeloma	Intravenously	C57BL/6	LCL161 smac-mimetics compound	The survival rate was 75% and posible acquired immunity agains re challenging with Vk12598 cells	
Retro-orbital/systemic	*vMyx-hTNF-GFP*	Naked	Multiple myeloma	Intravenously	C57BL/7	mono	No data	68
Retro-orbital/systemic	*vMyx-hTNF-GFP*	Bone marrow leukocytes	Multiple myeloma	Intravenously	C57BL/9	mono	The disease was deleyed, survival rates increased, the BM ex vivo loaded virus had superior therapeutic effects compared to the free virus	
Retro-orbital/systemic	*vMyx-hTNF-GFP*	Bone marrow leukocytes	Multiple myeloma	Intravenously	C57BL/10	cyclophosphamide	There was no significant difference in the deleay of the disease between the group treated only with cyclophosphamide	
Retro-orbital/systemic	*vMyx-hTNF-GFP*	Bone marrow leukocytes	Multiple myeloma	Intravenously	C57BL/11	LCL161 smac-mimetics compound	The survival rate was 60% posible acquired immunity agains re challenging with Vk12598 cells	
Retro-orbital/systemic	*vMyx-mLIGhT*	Peripheral blood mononuclear	metastasic lung cancer	Intravenously	BALB/CJ	Monotherapy	Highest survival rate, reduction of tumor burden in later stage model	
Retro-orbital/systemic	*vMyx-mLIGhT*	Peripheral blood mononuclear	metastasic lung cancer	Intravenously	BALB/CJ	anti-PD1	Highest survival rate, in some cases tumor regression, reduction of tumor burden in later stage model, better combined with ICI	79
Retro-orbital/systemic	*vMyx-mLIGhT*	Peripheral blood mononuclear cells	metastasic lung cancer	Intravenously	BALB/CJ	anti-PD1	Lower survival rate compared with the other treatments, its therapeutic benefit is lost in later stages of advanced disease model.	
Systemic	*vMyxFluc-tdTr*	bone marrow derived mesenchymal stem cells	Melanoma	Intravenously	C57BL/6		The foci number of Melanoma in the lungs of mice was small, bioluminescence in lungs peaked within minutes and faded away within 4 days, it was also high in the livers of +MEL and -MEL mice (with Melanoma lesions growing in lungs for 9 days or unchallenged mice)	
Systemic	*vMyxFluc-tdTr*	Naked	Melanoma	Intravenously	C57BL/7		The foci number of Melanoma in the lungs of mice was small, bioluminescence in lungs was not detected but it was present in the liver and spleen but faded away.	
Systemic	*vMyxIL15Rα-tdTr*	bone marrow derived mesenchymal stem cells	Melanoma	Intravenously	C57BL/8	Monotherapy	2 fold reduction compared to the unshielded virus group, extension of survival when administered three times. The NK cell percentage did not differ from controls significantly. At 48h the NK percentage in the lungs doubled which may be associated with transfer of virus between MSCs and Melanoma cells. CD4+ unchanged after 3 weeks in blood and lungs, CD8+ cells were present at similar percentages in blood, an increase in 2.5 fod of CD8+ T cells in lung tissue. CD3+,CD4+ Cd8+ in blood remained unchanged, CD4+ was decreased and CD8+ increased	65

Systemic	*vMyxIL15Rα-tdTr*	Naked	Melanoma	Intravenously	C57BL/9		Less reduction compared to the shielded virus, there is an early immune response seenin the NK cells that appear at 24 hours, it was higher in 2.8 fold compared to the shielded virus, but there was a disminution after 48h not differing from the shielded. An increase in 2.5 fod of CD8+ T cells in lung tissue	
Intratumoral	*vMyxΔserp2*	Naked	Agressive Alveolar Rhabdomyosarcoma	Subcutaneously	B6.Cg-Foxn1nu/J	Monotherapy	Clinical signs of disease that necessitated euthanasia were less comon in mice rated with the virus, there was no spread of the virus to off-target organs	
Intratumoral	*vMyxΔserp2*	Naked	Agressive Alveolar Rhabdomyosarcoma	Subcutaneously	B6.Cg-Foxn1nu/J	Oclacitinib	Clinical signs of disease that necessitated euthanasia were less comon in mice rated with the virus, there was no spread of the virus to off-target organs. By day 7, average hemorrhage and neutrophil grades was decreased compared to the mono therapy, and animals that made the criteria to be euthanized, there was a higher nectrosis grade compared to mice treated with PBS, but this didnt altered tumor volume, tumor growth rate or median survival. The viral replication was prolonged with oclacitinib	70
Intratumoral	*vMyx-IL15Rα-tdTr*	Naked	Glioma	Intracranial	C57BL/6J	Monotherapy	Increase of tumor infiltrated Ly-49G2+NK cells compared to the other virus.	
Intratumoral	*vMyx-IL15Rα-tdTr*	Naked	Glioma	Intracranial	C57BL/6J	rampamycin+ACT (trivax)	Had a better survival rate than the mice treated with PBS and celecovib an rampamycin	73
Intratumoral	*vMyx-IL15Rα-tdTr*	Naked	Glioma	Intracranial	C57BL/6J	rampamycin+ celecoxib + ACT (trivax)	Had the higher survival rate compared to the other treatment, there was an increase f the infiltrated CD8+T cells and a significant decrease of infiltrated Foxp3+Treg, there was an increased number of GARC-1-specific CD8+T cells, multiple apoptotic cells were present.	
Intratumoral	*vMyx-NDV-F*	Naked	Lewis Lung Carcinoma	Subcutaneously	C57/Bl6			
Intratumoral	*vMyx-RSV-F*	Naked	Lewis Lung Carcinoma	Subcutaneously	C57/Bl6 and NOD/Scid	Monotherapy	The treatments didnot reduce tumor burden and prolonged survival, instead increased overall tumor burden and reduced overall survival. The titer was lower with this virus because the construct was highly fusogenic	66
Intratumoral	*vMyx-NV-F*	Naked	Lewis Lung Carcinoma	Subcutaneously	C57/Bl6 and NOD/Scid			
Intratumoral	*vMyx-BPV-F*	Naked	Lewis Lung Carcinoma	Subcutaneously	C57/Bl6 and NOD/Scid			
